# Weak and Strong Type $A_1$–$A_\infty$ Estimates for Sparsely Dominated Operators

**DOI:** 10.1007/s12220-018-9989-2

**Published:** 2018-02-06

**Authors:** Dorothee Frey, Zoe Nieraeth

**Affiliations:** Delft Institute of Applied Mathematics, Delft University of Technology, P.O. Box 5031, 2600 GA Delft, The Netherlands

**Keywords:** Sparse domination, Muckenhoupt weights, Sharp weighted bounds, 42B20, 42B25

## Abstract

We consider operators *T* satisfying a sparse domination property

$$\begin{aligned} |\langle Tf,g\rangle |\le c\sum _{Q\in \mathscr {S}}\langle f\rangle _{p_0,Q}\langle g\rangle _{q_0',Q}|Q| \end{aligned}$$

with averaging exponents $1\le p_0<q_0\le \infty$. We prove weighted strong type boundedness for $p_0<p<q_0$ and use new techniques to prove weighted weak type $(p_0,p_0)$ boundedness with quantitative mixed $A_1$–$A_\infty$ estimates, generalizing results of Lerner, Ombrosi, and Pérez and Hytönen and Pérez. Even in the case $p_0=1$ we improve upon their results as we do not make use of a Hörmander condition of the operator *T*. Moreover, we also establish a dual weak type $(q_0',q_0')$ estimate. In a last part, we give a result on the optimality of the weighted strong type bounds including those previously obtained by Bernicot, Frey, and Petermichl.

## Introduction

In recent years, after a solution was found to the well-known $A_2$ conjecture [24], the role of sparse operators has become increasingly important in the weighted theory of many operators, see for instance [5, 12, 14, 29, 31] and references therein. Sparse domination yields optimal quantitative $A_p$ estimates for $1<p<\infty$, for example, for the classical Riesz transforms in $\mathbf {R}^n$. As has been shown by Bernicot, Frey, and Petermichl [6], the idea of sparse domination reaches far beyond the theory of Calderón–Zygmund operators. Indeed, one can consider the Riesz transform $\nabla L^{-1/2}$ in, e.g. a convex doubling domain in $\mathbf {R}^n$, where *L* is the Laplace operator with respect to Neumann boundary conditions. Generally, the Riesz transform in such a setting does not satisfy any pointwise regularity estimates and therefore falls outside of the class of Calderón–Zygmund operators. However, it satisfies a sparse domination property which does in fact yield the quantitative weighted bounds from the $A_2$ conjecture. In $\mathbf {R}^n$, foregoing the full range of $1<p<\infty$, one can consider the Riesz transform for elliptic operators $L=-{{\mathrm{div}}}(A\nabla )$ for *A* with bounded, complex coefficients. Such operators are only bounded in $L^p$ for a certain range $p_0<p<q_0$, and it was established in [6] that they satisfy a sparse domination property

$$\begin{aligned} |\langle Tf,g\rangle |\le c\sum _{Q\in \mathscr {S}}\langle f\rangle _{p_0,Q}\langle g\rangle _{q_0',Q}|Q| \end{aligned}$$

from which general quantitative weighted bounds in the respective weighted $L^p$-spaces are deduced.

For Calderón–Zygmund operators, weighted weak type (1, 1) estimates were established by Lerner et al. [33] and later improved upon by Hytönen and Pérez [26]. In this article, we establish the corresponding $(p_0,p_0)$ estimate in the more general setting described above. The arguments used in [33] rely on introducing weights in the classical arguments involving Calderón–Zygmund decompositions $f=g+b$ and the vanishing mean value property of the ‘bad’ part *b* in combination with the Hörmander condition of the kernel of the operator. In general, the operators we are considering here need not be integral operators at all and for the more general operators such as the Riesz transform associated to an elliptic operator, an argument by Blunck and Kunstmann [8] (see also [23]) gave a weak type $(p_0,p_0)$ boundedness using an adapted $L^{p_0}$ Calderón–Zygmund decomposition, where a certain cancellation of the operator with respect to the semigroup generated by the elliptic operator replaces the regularity estimates of the kernel. Weights were then introduced into this argument by Auscher and Martell [2], but it seems like these techniques do not yield optimal bounds in terms of the constants of the weights. Therefore, we give a new argument to establish the corresponding bounds while still recovering the old bounds found in [26].

Here, in order to combine the previous approaches and to tie the theory together, we deduce quantitative weighted bounds directly from sparse domination assumptions. We introduce weights into a weak boundedness argument for sparse operators where there exists a Calderón–Zygmund decomposition with the property that the ‘bad’ part *b* cancels completely. We then combine this with generalizations of the main lemmata used in [33]. Moreover, we leave the Euclidean setting and extend the results to more general doubling metric measure spaces including certain bounded domains and Riemannian manifolds as was also studied in [8] and [2, 3].

In a last part we show that the strong type weighted estimates are optimal, given a precise control of the asymptotic behaviour of the unweighted $L^p$ operator norm of *T* at the endpoints $p=p_0$ and $p=q_0$. We give an example of such an operator in the case $p_0=1$, $q_0=n$.

### The Setting

We consider the Euclidean space $\mathbf {R}^n$ equipped with a Borel measure $\mu$ that satisfies $0<\mu (B)<\infty$ for all balls *B* and which satisfies the doubling property, i.e. there is a $C>0$ such that

1.1 $$\begin{aligned} \mu (2B)\le C\mu (B) \end{aligned}$$

for all balls *B*, where 2*B* denotes the ball with the same centre as *B* and whose radius is twice that of the radius of *B*. Taking the smallest such *C* we define $\nu :=\log _2 C$, which we refer to as the *doubling dimension*. We write $|E|:=\mu (E)$ and for each measurable set *E* of finite non-zero measure and each $0<p\le \infty$ we will write

$$\begin{aligned} \langle f\rangle _{p,E}:=\Vert f\chi _E\Vert _{L^p(|E|^{-1}\mathrm {d}\mu )}, \end{aligned}$$

where $\chi _E$ denotes the indicator function of the set *E*. We write $\langle f,g\rangle :=\int f g\,\mathrm {d}\mu$, and define $p'=p/(p-1)\in [1,\infty ]$ for $1\le p\le \infty$.

For $\alpha \in \big \{0,\frac{1}{3},\frac{2}{3}\big \}^n$ we will consider the translated dyadic systems

$$\begin{aligned} \mathscr {D}^\alpha :=\bigcup _{k\in \mathbf {Z}}\big \{2^{-k}\big ([0,1)^n+m+(-1)^k\alpha \big ):m\in \mathbf {Z}^n\big \}, \end{aligned}$$

and $$\mathscr {D}:=\cup _\alpha \mathscr {D}^\alpha $$.

The collection $\mathscr {D}$ is used as a replacement for the collection of balls or the collection of all cubes in $\mathbf {R}^n$, which is justified by the fact that for any ball $B(x;r)\subseteq \mathbf {R}^n$ there is a cube $Q\in \mathscr {D}$ so that $B(x;r)\subseteq Q$ and ${{\mathrm{diam}}}(Q)\le \rho r$ for a constant $\rho =\rho (n)>0$, and for any cube $P\subseteq \mathbf {R}^n$ there is a cube $Q\in \mathscr {D}$ such that $P\subseteq Q$ and $\ell (Q)\le 6\ell (P)$, where $\ell (R)$ denotes the side length of a cube *R*.

We say that a collection $\mathscr {S}\subseteq \mathscr {D}$ is called $\eta$-*sparse* for $0<\eta \le 1$ if for each $\alpha \in \big \{0,\frac{1}{3},\frac{2}{3}\big \}^n$ there is a pairwise disjoint collection $(E_Q)_{Q\in \mathscr {S}\cap \mathscr {D}^\alpha }$ of measurable sets so that $E_Q\subseteq Q$ and $|Q|\le \eta ^{-1}|E_Q|$.

#### Remark 1.1

Since $\mathbf {R}^n$ is connected and unbounded, the doubling property implies that $\mu (\mathbf {R}^n)=\infty$ [21]. We are working in $\mathbf {R}^n$ for notational reasons only; since our applications lie in a more general framework, our arguments are written so that they work with minimal adaptations in general doubling metric measure spaces *X*. Our main results remain true even when $\mu (X)<\infty$, for example when *X* is a bounded Lipschitz domain in $\mathbf {R}^n$. We will detail how this can be seen in Sect. 4.

We let $\mathcal {D}$ be a space of test functions on $\mathbf {R}^n$ with the property that it is dense in $L^p(w)$ for all $1\le p<\infty$ and all weights $w\in A_\infty$, for example, $\mathcal {D}=C_c^\infty (\mathbf {R}^n)$.

#### Definition 1.2

Let *T* be a (sub)linear operators, initially defined on $\mathcal {D}$, with the following property: There are $1\le p_0<q<q_0\le \infty$ and constants $c>0$ and $0<\eta \le 1$ so that for each pair of functions $f,g\in \mathcal {D}$ there is an $\eta$-sparse collection $\mathscr {S}\subseteq \mathscr {D}$ so that

1.2 $$\begin{aligned} |\langle Tf,g\rangle |\le c\sum _{Q\in \mathscr {S}}\langle f\rangle _{p_0,Q}\langle g\rangle _{q_0',Q}|Q|. \end{aligned}$$

Then we will write $T\in S(p_0,q_0)$, or $T\in S(p_0,q_0;\mu )$ if we wish to emphasize the underlying measure, and we shall refer to the operators in this class as *sparsely dominated operators*.

If $T\in S(p_0,q_0)$, then it extends to a bounded operator on $L^p$ for all $p_0<p<q_0$, see Proposition 2.2. For examples of operators in this class we refer the reader to Sect. 1.3

When writing that a constant $C=C(T)>0$ depends on *T*, we mean that it depends on the constants $c,\eta$ in the domination property (1.2). We remark that the sum on the right-hand side of (1.2) can be split into $3^n$ sums by considering the different dyadic grids, simplifying the proofs by only having to consider a single dyadic grid at a time. Finally, we remark that if *T* is linear, then $T\in S(p_0,q_0)$ if and only if $T^*\in S(q_0',p_0')$, where $T^*$ denotes the dual operator of *T*.

We will write $A\lesssim B$ when there is a constant $C>0$, independent of the important parameters, so that $A\le C B$. Moreover we write $A\simeq B$ if $A\lesssim B$ and $B\lesssim A$.

### Main Results

For $1\le p_0<q_0\le \infty$ we consider an operator $T\in S(p_0,q_0)$. Then *T* will be of strong type (*p*, *p*) for any $p_0<p<q_0$ and of weak type $(p_0,p_0)$, see Proposition 2.2. As a matter of fact, *T* will satisfy weighted boundedness for various classes of weights. It has been shown in [6] that for $p_0<p<q_0$ and any $w\in A_{p/p_0}\cap {{\mathrm{RH}}}_{(q_0/p)'}$ we have

1.3 $$\begin{aligned} \Vert T\Vert _{L^p(w)\rightarrow L^p(w)}\lesssim & {} \left[ w^{(q_0/p)'}\right] _{A_{\phi (p)}}^{\max \left( \frac{1}{p-p_0},\frac{q_0-1}{q_0-p}\right) \big /\left( \frac{q_0}{p}\right) '}\nonumber \\\le & {} \left( [w]_{A_{p/p_0}}[w]_{{{\mathrm{RH}}}_{(q_0/p)'}}\right) ^{\max \left( \frac{1}{p-p_0}, \frac{q_0-1}{q_0-p}\right) }, \end{aligned}$$

where $\phi (p)=(q_0/p)'(p/p_0-1)+1$, and that the exponent in the last estimate is optimal for sparse operators. This generalizes the positive result of the well-known $A_2$-conjecture, stating that for all Calderón–Zygmund operators *T* one has

1.4 $$\begin{aligned} \Vert T\Vert _{L^p(w)\rightarrow L^p(w)}\lesssim [w]_{A_p}^{\max \left( \frac{1}{p-1},1\right) }. \end{aligned}$$

Indeed, the result in (1.3) recovers this result since Calderón–Zygmund operators are in the class $S(1,\infty )$. Historically, the estimate (1.4) was first proven to be true for the Beurling–Ahlfors transform by Petermichl and Volberg [38], solving an optimal regularity problem for solutions to Beltrami equations. In between this period and the time that (1.4) was established in full generality by Hytönen [24], it was shown by Lerner, Ombrosi, and Pérez [32] that for all Calderón–Zygmund operators *T* one has

1.5 $$\begin{aligned} \Vert T\Vert _{L^p(w)\rightarrow L^p(w)}\lesssim p p'\log \left( e+\frac{1}{p-1}\right) [w]_{A_1} \end{aligned}$$

for all $1<p<\infty$, showing a significantly better exponent of the constant of the weight when considering the smaller class of weights $A_1\subseteq A_p$. Using mixed $A_1$–$A_\infty$ type estimates, this result was improved by Hytönen and Pérez [26] to

1.6 $$\begin{aligned} \Vert T\Vert _{L^p(w)\rightarrow L^p(w)}\lesssim pp'[w]_{A_\infty }^{\frac{1}{p'}}[w]^{^{\frac{1}{p}}}_{A_1}, \end{aligned}$$

for $1<p<\infty$, where they are considering Wilson’s $A_\infty$ constant

$$\begin{aligned}{}[w]_{A_\infty }=\sup _{B \text { a ball }}\frac{1}{w(B)}\int _B\!\mathscr {M}(w\chi _B)\,{\mathrm {d}}\mu , \end{aligned}$$

which appears in [41–43]. They also provided an improvement to (1.4) using mixed $A_p$–$A_\infty$ type estimates. Such mixed type estimates have also appeared in the recent work by Li [34], who gives a direct improvement of (1.3).

To continue on along this line of results, we establish the following:

#### Theorem 1.3

Let $1\le p_0<p<q_0\le \infty$, $T\in S(p_0,q_0)$, and $w\in A_1\cap {{\mathrm{RH}}}_{(q_0/p)'}$. Then there is a constant $c=c(T,\nu ,n)>0$ so that

1.7 $$\begin{aligned} \Vert T\Vert _{L^p(w)\rightarrow L^p(w)}\le c c_p\left[ w^{(q_0/p)'}\right] _{A_\infty }^{\frac{1}{p'}}\left[ w^{(q_0/p)'}\right] ^{^{\frac{1}{p(q_0/p)'}}}_{A_1}, \end{aligned}$$

with

$$\begin{aligned} c_p=\left[ \left( \frac{p'}{q_0'}\right) '\right] ^ {\frac{1}{q_0'}}\left[ \left( \frac{p_0'}{p'}\right) '\left( \frac{p}{p_0} \right) '\right] ^{\frac{1}{p_0}}. \end{aligned}$$

In particular, we have

1.8 $$\begin{aligned} \Vert T\Vert _{L^p(w)\rightarrow L^p(w)}\lesssim c_p\left[ w^{(q_0/p)'}\right] _{A_1}^{\frac{1}{q_0'}}\le c_p\left( [w]_{A_1}[w]_{{{\mathrm{RH}}}_{(q_0/p)'}}\right) ^{\frac{q_0-1}{q_0-p}}. \end{aligned}$$

Our result (1.7) recovers (1.6) when setting $p_0=1$, $q_0=\infty$. One shows that (1.8) follows from (1.7) by applying (2.1) and Proposition 2.1(ii). This result recovers the exponent in (1.5) when $q_0=\infty$.

The constants found in the estimate (1.7) can be used to establish weighted weak type $(p_0,p_0)$ boundedness. In the work of Lerner, Ombrosi, and Pérez [32] it was shown that for all Calderón–Zygmund operators *T* and all weights $w\in A_1$ one has

1.9 $$\begin{aligned} \Vert T\Vert _{L^1(w)\rightarrow L^{1,\infty }(w)}\lesssim [w]_{A_1}\log (e+[w]_{A_1}). \end{aligned}$$

This result is related to the weak Muckenhoupt–Wheeden conjecture, which is now known to be false [36], stating that one has linear dependence on $[w]_{A_1}$ on the right-hand side of (1.9), and the logarithm can be removed. However, the result (1.9) was improved by Hytönen and Pérez [26] to

1.10 $$\begin{aligned} \Vert T\Vert _{L^1(w)\rightarrow L^{1,\infty }(w)}\lesssim [w]_{A_1}\log (e+[w]_{A_\infty }). \end{aligned}$$

It is expected that this dependence on the constants of the weight is optimal.

Both the proofs of (1.9) and (1.10) rely on taking a Calderón–Zygmund decomposition $f=g+b$. Here, the Hörmander condition of the kernel of *T* is used to deal with the ‘bad’ part *b*, using an argument that can already be found in [37] (namely, they use [18, Lemma 3.3, p. 413]). Since we are making no such assumptions on our operators, which may not even be integral operators, we rely on new methods to deal with this term, using only sparse domination. We establish the following result:

#### Theorem 1.4

Let $1\le p_0<p<q_0\le \infty$, $T\in S(p_0,q_0)$, and $w\in A_1\cap {{\mathrm{RH}}}_{(q_0/p_0)'}$. Then there is a constant $c=c(T,p_0,q_0,\nu ,n)>0$ so that

$$\begin{aligned} \Vert T\Vert _{L^{p_0}(w)\rightarrow L^{p_0,\infty }(w)}\le c\psi (w) \end{aligned}$$

with

$$\begin{aligned} \psi (w)={\left\{ \begin{array}{ll} {[}w]_{A_1}\log (e+[w]_{A_\infty }) &{} \mathrm{{if}}\;p_0=1,\, q_0=\infty ;\\ {[}w]^{\frac{1}{p_0}}_{A_1}[w]^{\frac{1}{p_0'}}_{A_\infty } \log \left( e+[w]_{A_\infty }\right) ^{\frac{2}{p_0}} &{} \mathrm{{if}}\;p_0>1,\, q_0=\infty ;\\ \left[ w^{q_0'}\right] _{A_\infty }[w]_{A_1}[w]_{{{\mathrm{RH}}}_{q_0'}} &{} \mathrm{{if}}\;p_0=1,\,q_0<\infty ;\\ \left[ w^{(q_0/p_0)'}\right] ^{1+\frac{1}{p_0}}_{A_\infty }\left( [w]_{A_1}[w]_{{{\mathrm{RH}}}_{(q_0/p_0)'}}\right) ^{\frac{1}{p_0}} &{} \mathrm{{if}}\;p_0>1,\, q_0<\infty . \end{array}\right. } \end{aligned}$$

We note that in particular we recover the bound (1.10). It is of interested to point out that we get this bound even for operators outside of the class of Calderón–Zygmund operators that are in $S(1,\infty )$, see Example 1.8.

We also establish a dual result of the type first studied in [33], generalizing the result [26, Theorem 1.23]. Here we denote by $T^*$ the dual operator of *T* for linear *T*.

#### Theorem 1.5

Let $1\le p_0<q_0\le \infty$, $T\in S(p_0,q_0)$ linear and $w\in A_1$. Then there is a constant $c=c(T,p_0,q_0,\nu ,n)>0$ so that

$$\begin{aligned} \left\| \frac{T^*f}{w^{\frac{1}{q_0'}}}\right\| _{L^{q_0',\infty }(w)}\le c\left( [w]_{A_\infty }\log \left( e+[w]_{A_1}\right) \right) ^{\frac{1}{q_0'}}\Vert f\Vert _{q_0'} \end{aligned}$$

for all $$f\in L^{q_0'}$$.

Using the ideas of [35], we then establish optimality of the weighted estimates in terms of the asymptotic behaviour of the unweighted $L^p$ operator norm of *T* at the endpoints $p=p_0$ and $p=q_0$. We refer to Definition 5.1 for the definition of the exponents $\alpha _T(p_0)$ and $\gamma _T(q_0)$.

#### Theorem 1.6

Let $1\le p_0<q_0\le \infty$, let $T\in S(p_0,q_0)$, and let $w\in A_{p/p_0}\cap {{\mathrm{RH}}}_{(q_0/p)'}$. Then the exponent in the estimate

$$\begin{aligned} \Vert T\Vert _{L^p(w) \rightarrow L^p(w)}\lesssim \left[ w^{(q_0/p)'}\right] _{A_{\phi (p)}}^{\max \left( \frac{1}{p-p_0},\frac{q_0-1}{q_0-p}\right) \big /\left( \frac{q_0}{p}\right) '} \end{aligned}$$

from [6] is optimal under the assumption that $\alpha _T(p_0)=1/p_0$ and $\gamma _T(q_0)=1/q_0'$.

Moreover, for $w\in A_1\cap {{\mathrm{RH}}}_{(q_0/p)'}$, the exponent in the estimate

$$\begin{aligned} \Vert T\Vert _{L^p(w)\rightarrow L^p(w)}\lesssim \left[ w^{(q_0/p)'}\right] _{A_1}^{\frac{1}{q_0'}} \end{aligned}$$

from Theorem 1.3 is optimal under the assumption that $\gamma _T(q_0)=1/q_0'$.

In the example of the Riesz transform on two copies of $\mathbf {R}^n$ glued smoothly along their unit circles [9], it is known that $q_0=n$ and $\gamma _T(q_0)=(n-1)/n$, and thus the weighted estimate is optimal. See Example 5.5.

### Examples

There is a wealth of examples of sparsely dominated operators. Other than the class of Calderón–Zygmund operators, our main examples can be found in [6, Sect. 3]. See also the earlier work [2]. We point out several examples of particular interest here.

#### Example 1.7

(Riesz transform associated with elliptic second-order divergence form operators). Let *A* be a complex, bounded, measurable matrix-valued function in $\mathbf {R}^n$ satisfying the ellipticity condition ${{\mathrm{Re}}}(A(x)\xi \cdot \overline{\xi })\ge \lambda |\xi |^2$ for all $\xi \in \mathbf {C}^n$ and a.e. $x\in \mathbf {R}^n$. Then one can define a maximal accretive operator

$$\begin{aligned} Lf:=-{{\mathrm{div}}}(A\nabla f) \end{aligned}$$

which generates a semigroup $(e^{-tL})_{t>0}$. If both the semigroup and the family $(\sqrt{t}\nabla e^{-tL})_{t>0}$ satisfy $L^{p_0}$–$L^{q_0}$ off-diagonal estimates, then the Riesz transform $\mathscr {R}:=\nabla L^{-1/2}$ is in the class $S(p_0,q_0)$. In particular we point out that if we are using the Lebesgue measure in dimension $\nu =n=1$, we have $p_0=1$ and $q_0=\infty$ so that $\mathscr {R}\in S(1,\infty )$. We refer the reader to [1] for more values of $p_0$ and $q_0$ in other dimensions in the Euclidean setting and to [6] for details on the sparse domination result.

#### Example 1.8

(Riesz transform associated to Neumann Laplacian) Suppose $\Delta$ is the Laplace operator associated with Neumann boundary conditions in a bounded convex doubling domain in $\mathbf {R}^n$. As studied in [40], the Riesz transform $\nabla \Delta ^{-1/2}$ will not in general have a kernel satisfying pointwise regularity estimates and is thus not in the class of Calderón–Zygmund operators. However, this operator does belong to the class $S(1,\infty )$ and will therefore satisfy the bound (1.6). Note that for this example we need to apply our results to a metric measure space other than $\mathbf {R}^n$. We refer the reader to Sect. 4 for an overview of the theory in bounded domains.

#### Example 1.9

(Fourier multipliers) Let *m* be the function in $\mathbf {R}^n$ defined by $m(\xi )=1-|\xi |^2$ for $|\xi |\le 1$ and $m(\xi )=0$ elsewhere. For $\delta \ge 0$, the Bochner–Riesz operator $\mathcal {B}^\delta$ is defined as the Fourier multiplier $\mathcal {B}^\delta f:=(m^\delta \hat{f})^{\vee }$. Then, for any $\delta >0$ there exists a $1<p_0<2$ so that for any $0<\varepsilon <2-p_0$ we have $\mathcal {B}^\delta \in S(p_0+\varepsilon , 2)$. For details we refer the reader to [5].

## Preliminaries

### Notation

For $1\le p<\infty$ we denote by $\mathscr {M}_p$ the uncentred dyadic maximal operator

$$\begin{aligned} \mathscr {M}_pf:=\sup _{Q\in \mathscr {D}^\alpha }\langle f\rangle _{p,Q}\chi _Q, \end{aligned}$$

where it will be made clear from the context which dyadic grid $\mathscr {D}^\alpha$ we are considering, and where we will write $\mathscr {M}:=\mathscr {M}_1$. Similarly we define $\mathscr {M}_p^{\mathscr {B}}$ and $\mathscr {M}^{\mathscr {B}}$ to be the uncentred maximal operators with respect to balls rather than cubes.

We list some of the basic definitions and facts about weights. A measurable function $w:\mathbf {R}^n\rightarrow (0,\infty )$ is called a weight. We identify a weight *w* with a Borel measure by setting

$$\begin{aligned} w(E):=\int _E\!w\,\mathrm {d}\mu \end{aligned}$$

for all measurable sets $E\subseteq \mathbf {R}^n$. For $1\le p\le \infty$ we, respectively, denote by $L^p(w)$ and $L^{p,\infty }(w)$ the Lebesgue and weak Lebesgue spaces with measure *w*.

For $1\le p<\infty$ we say that $w\in A_p$ if

$$\begin{aligned}{}[w]_{A_p}:=\sup _{Q\in \mathscr {D}}\langle w\rangle _{1,Q}\langle w^{-1}\rangle _{p'-1,Q}<\infty , \end{aligned}$$

where for $p=1$ we use the limiting interpretation $\langle w^{-1}\rangle _{p'-1,Q}=\langle w^{-1}\rangle _{\infty ,Q}=({{\mathrm{ess\,inf}}}_Q w)^{-1}$. We say that $w\in A_\infty$ if its Wilson $A_\infty$ constant is finite, that is, if

$$\begin{aligned}{}[w]_{A_\infty }:=\sup _{B\text { a ball }}\frac{1}{w(B)}\int _B\!\mathscr {M}^{\mathscr {B}}(w\chi _B)\,\mathrm {d}\mu <\infty , \end{aligned}$$

where the supremum is taken over all balls $B\subseteq \mathbf {R}^n$. For an overview of this constant we refer the reader to [27] and references therein. In particular we point out that for a dimensional constant $c=c(n,\nu )>0$ we have

2.1 $$\begin{aligned} c[w]_{A_\infty }\le [w]_{A_p}\le [w]_{A_q},\quad 1\le q<p<\infty , \end{aligned}$$

where the first inequality here can be found in [26, Proposition 2.2], while the second one follows from Hölder’s inequality.

For $1<s\le \infty$ we say that $w\in {{\mathrm{RH}}}_s$ if

$$\begin{aligned}{}[w]_{{{\mathrm{RH}}}_s}:=\sup _{Q\in \mathscr {D}}\langle w\rangle _{s,Q}\langle w\rangle ^{-1}_{1,Q}<\infty . \end{aligned}$$

For $s=1$ we will use the interpretation ${{\mathrm{RH}}}_1=A_\infty$, where we set $[w]_{{{\mathrm{RH}}}_1}:=1$.

We provide some facts about the classes $A_1$ and $A_\infty$ that we will use.

#### Proposition 2.1

(i) $A_q=\bigcup _{1\le p<q}A_p$ for $1<q\le \infty$ and ${{\mathrm{RH}}}_s=\bigcup _{s<r\le \infty }{{\mathrm{RH}}}_r$ for $1\le s<\infty$. In particular we have $w\in A_\infty$ if and only if $w\in A_p$ for some $1\le p<\infty$.
(ii) For $1\le p<\infty$, $1\le s<\infty$ we have $w\in A_p\cap {{\mathrm{RH}}}_s$ if and only if $w^s\in A_{s(p-1)+1}$. Moreover, we have
  $$\begin{aligned}{}[w^s]_{A_{s(p-1)+1}}\le \left( [w]_{A_p}[w]_{{{\mathrm{RH}}}_s}\right) ^s. \end{aligned}$$
(iii) There are constants $c,\kappa >0$ depending only on the doubling dimension $\nu$, so that for every $w\in A_1$ we have
  $$\begin{aligned} \mathscr {M}^{\mathscr {B}}_q w\le c[w]_{A_1}w\quad \text {for }1\le q\le 1+\frac{1}{\kappa [w]_{A_\infty }}. \end{aligned}$$

#### Proof

For (i) we refer the reader to [20, 41]. Property (ii) can be found in [28].

Property (iii) is a consequence of [27, Theorem 1.1]. Indeed, this result states that there are constants $c,\kappa >0$ depending only on $\nu$ such that for any ball *B* we have $\langle w\rangle _{q(w),B}\le c\langle w\rangle _{1,2B}$, where $q(w):=1+1/(\kappa [w]_{A_\infty })$. Thus, (iii) follows from Hölder’s inequality and the definition of $A_1$. $\square$

### Weighted Boundedness of Sparsely Dominated Operators

We wish to give some heuristic arguments as to why we can expect certain weighted boundedness of sparsely dominated operators. We start with the following observation:

#### Proposition 2.2

Let $1\le p_0<q_0\le \infty$ and $T\in S(p_0,q_0)$. Then *T* is of strong type (*p*, *p*) for all $p_0<p<q_0$ and of weak type $(p_0,p_0)$.

The verification of the strong boundedness is by now standard, see also [13]. While the weak type boundedness should be well known, we could not find a precise reference for the cases where $p_0>1$. For the case $p_0=1$ we refer the reader to [12, Theorem E], see also [4, Proposition 6]. For completeness we give a proof of the general case here, which we defer to the end of this section.

We will show that if an operator *T* lies in $S(p_0,q_0;\mu )$, then *T* must also lie in $S(q_-,q_+;w)$ for appropriate weights *w*, and for certain $q_-<q_+$ depending on *w*. Then Proposition 2.2 implies that *T* satisfies weighted boundedness.

First we note that if we have a sparse collection $\mathscr {S}\subseteq \mathscr {D}$ with respect to the reference measure $\mu$, then $\mathscr {S}$ is also sparse with respect to all weights $w\in A_\infty$. Indeed, suppose $w\in A_p$ for some $1\le p<\infty$ and suppose $\mathscr {S}$ is $\eta$-sparse with $(E_Q)_{Q\in \mathscr {S}\cap \mathscr {D}^\alpha }$ as one of the associated pairwise disjoint collections. Then, by Hölder’s inequality (use the first equation in (2.2) with $f=\chi _{E_Q}$, $p=1$),

$$\begin{aligned} w(Q)\le \left( \frac{|Q|}{|E_Q|}\right) ^p[w]_{A_p}w(E_Q)\le \eta ^{-p}[w]_{A_p}w(E_Q). \end{aligned}$$

Hence, $\mathscr {S}$ is $[w]_{A_p}^{-1}\eta ^p$-sparse with respect to the measure *w* with the same collections $(E_Q)_{Q\in \mathscr {S}\cap \mathscr {D}^\alpha }$.

Next we observe that for any $1\le p\le q<\infty$ it follows from Hölder’s inequality that

2.2 $$\begin{aligned} \begin{aligned} \langle f\rangle _{p,Q}&\le [w]_{A_{q/p}}^{\frac{1}{q}}\left( \frac{1}{w(Q)}\int _Q\!|f|^q w\,\mathrm {d}\mu \right) ^{\frac{1}{q}},\\ \langle g w\rangle _{q',Q}|Q|&\le [w]_{{{\mathrm{RH}}}_{(q/p)'}}^{\frac{1}{p}}\left( \frac{1}{w(Q)}\int _Q\!|g|^{p'} w\,\mathrm {d}\mu \right) ^{\frac{1}{p'}}w(Q). \end{aligned} \end{aligned}$$

Thus, if $T\in S(p_0,q_0;\mu )$ and $w\in A_{p_1/p_0}\cap {{\mathrm{RH}}}_{(q_0/q_1)'}$ for some $p_0< p_1\le q_1<q_0$, then it follows from the self-improvement properties (i) of Proposition 2.1 that we can find $p_0\le q_-< p_1$, $q_1<q_+\le q_0$ so that $w\in A_{q_-/p_0}\cap {{\mathrm{RH}}}_{(q_0/q_+)'}$. Picking appropriate functions *f*, *g*, and by applying the sparse domination property to the pair *f*, *gw*, we find a sparse collection $\mathscr {S}\subseteq \mathscr {D}$ so that by (2.2) we have

$$\begin{aligned} \left| \int (Tf)gw\,\mathrm {d}\mu \right| \lesssim \sum _{Q\in \mathscr {S}}\left( \frac{1}{w(Q)}\int _Q\!|f|^{q_-} w\,\mathrm {d}\mu \right) ^{\frac{1}{q_-}}\left( \frac{1}{w(Q)}\int _Q\!|g|^{q_+'} w\,\mathrm {d}\mu \right) ^{\frac{1}{q_+'}}w(Q). \end{aligned}$$

In other words, we have $T\in S(q_-,q_+;w)$ and thus we obtain the boundedness

$$\begin{aligned} T:L^p(w)\rightarrow L^p(w),\quad w\in A_{p_1/p_0}\cap {{\mathrm{RH}}}_{(q_0/q_1)'},\quad p_1\le p\le q_1. \end{aligned}$$

For the case where $p_1=q_1=p_0$ and thus when $w\in A_1\cap {{\mathrm{RH}}}_{(q_0/p_0)'}$, an analogous reasoning shows that for some $p_0<q_+$ we have $T\in S(p_0,q_+;w)$. Hence, it follows from Proposition 2.2 that *T* is of weak type $(p_0,p_0)$ with respect to such weights.

Our main results deal with the cases $p_1=p_0$ where we establish quantitative bounds of *T* in terms of the characteristic constants of the weight in the situations

$$\begin{aligned} \begin{aligned} T:L^p(w)\rightarrow L^p(w),&\quad w\in A_1\cap {{\mathrm{RH}}}_{(q_0/p)'},\\ T:L^{p_0}(w)\rightarrow L^{p_0,\infty }(w),&\quad w\in A_1\cap {{\mathrm{RH}}}_{(q_0/p_0)'}. \end{aligned} \end{aligned}$$

#### Proof of Proposition 2.2

By splitting into $3^n$ terms, we may assume without loss of generality that our sparse domination occurs in a single dyadic grid $\mathscr {D}^\alpha$ throughout our arguments.

Let $p_0<p<q_0$ and let $f\in L^p\cap \mathcal {D}$, $g\in L^{p'}\cap \mathcal {D}$. Then we can find a sparse collection $\mathscr {S}\subseteq \mathscr {D}^\alpha$ so that

$$\begin{aligned} |\langle Tf,g\rangle |\lesssim & {} \sum _{Q\in \mathscr {S}}\langle f\rangle _{p_0,Q}\langle g\rangle _{q_0',Q}|Q|\lesssim \sum _{Q\in \mathscr {S}}{{\mathrm{ess\,inf}}}_Q\big (\mathscr {M}_{p_0}f\mathscr {M}_{q_0'}g\big )|E_Q|\\\lesssim & {} \int \mathscr {M}_{p_0}f\mathscr {M}_{q_0'}g\,\mathrm {d}\mu . \end{aligned}$$

By using Hölder’s inequality and by noting that $p>p_0$, $p'>q_0'$, it remains to observe that $\Vert \mathscr {M}_{p_0}f\Vert _p\lesssim \Vert f\Vert _p$ and $\Vert \mathscr {M}_{q_0'}g\Vert _{p'}\lesssim \Vert g\Vert _{p'}$. Hence, *T* extends to a bounded operator in $L^p$.

For the second assertion we will use the equivalence

2.3 $$\begin{aligned} \Vert T\Vert _{L^{p_0}\rightarrow L^{p_0,\infty }}\simeq \sup _{\Vert f\Vert _{p_0}=1}\sup _{\begin{array}{c} E\subseteq \mathbf {R}^n\\ 0<|E|<\infty \end{array}}\inf _{\begin{array}{c} E'\subseteq E\\ |E|\le 2|E'| \end{array}}\sup _{|h|\le \chi _{E'}}|E|^{\frac{1}{p_0}-1}|\langle Tf,h\rangle |, \end{aligned}$$

with $f,h\in \mathcal {D}$, see [19, Exercise 1.4.14]. Given such an *f* with $\Vert f\Vert _{p_0}=1$ and $E\subseteq \mathbf {R}^n$ of finite positive measure we define

$$\begin{aligned} \Omega :=\Big \{\mathscr {M}^{\mathscr {B}}(|f|^{p_0})>K|E|^{-1}\Big \},\quad E':=E\backslash \Omega , \end{aligned}$$

where *K* is chosen large enough to ensure that $|E|\le 2|E'|$. Let $h\in \mathcal {D}$ with $|h|\le \chi _{E'}$. Then we can find a sparse collection $\mathscr {S}\subseteq \mathscr {D}^\alpha$ such that

2.4 $$\begin{aligned} |\langle Tf,h\rangle |\lesssim \sum _{Q\in \mathscr {S}}\langle f\rangle _{p_0,Q}\langle h\rangle _{q_0',Q}|Q|=\sum _{\begin{array}{c} Q\in \mathscr {S}\\ Q\cap E'\ne \emptyset \end{array}}\langle f\rangle _{p_0,Q}\langle h\rangle _{q_0',Q}|Q|. \end{aligned}$$

We proceed by taking a Calderón–Zygmund decomposition of $|f|^{p_0}\in L^1$. We can find a disjoint collection $\mathscr {P}\subseteq \mathscr {D}^\alpha$ of cubes so that $\Omega =\cup _{P\in \mathscr {P}}P$ and functions *g*, $(b_P)_{P\in \mathscr {P}}$ so that $|f|^{p_0}=g+\sum _{P\in \mathscr {P}}b_P$ and

2.5 $$\begin{aligned}&{{\mathrm{supp}}}b_P\subseteq P,\quad \int _P\!b_P\,\mathrm {d}\mu =0, \end{aligned}$$

2.6 $$\begin{aligned}&\Vert g\Vert _\infty \lesssim |E|^{-1},\quad \Vert g\Vert _{1}\lesssim 1. \end{aligned}$$

Noting that for all $P\in \mathscr {P}$ we have $P\cap E'=\emptyset$, the properties of the dyadic system imply that for any $Q\in \mathscr {S}$ with $Q\cap E'\ne \emptyset$ we have $P\subseteq Q$ whenever $P\cap Q\ne \emptyset$. But then by (2.5) and arguments similar to the ones in the first part of the proof we have

2.7 $$\begin{aligned} \sum _{\begin{array}{c} Q\in \mathscr {S}\\ Q\cap E'\ne \emptyset \end{array}}\langle f\rangle _{p_0,Q}\langle h\rangle _{q_0',Q}|Q|= & {} \sum _{\begin{array}{c} Q\in \mathscr {S}\\ Q\cap E'\ne \emptyset \end{array}}\left( \frac{1}{|Q|}\int _Q\!g\,\mathrm {d}\mu \right) ^{\frac{1}{p_0}}\langle h\rangle _{q_0',Q}|Q|\nonumber \\\lesssim & {} \Vert |g|^{\frac{1}{p_0}}\Vert _q\Vert h\Vert _{q'}. \end{aligned}$$

Thus, by combining (2.4) and (2.7), we find using (2.6) that

$$\begin{aligned} |\langle Tf,h\rangle |\lesssim \left( \Vert g\Vert ^{1-\frac{p_0}{q}}_\infty \Vert g\Vert ^{\frac{p_0}{q}}_1\right) ^{\frac{1}{p_0}}\Vert h\Vert _\infty |E'|^{\frac{1}{q'}}\lesssim |E|^{\frac{1}{q}-\frac{1}{p_0}}|E|^{\frac{1}{q'}}=|E|^{1-\frac{1}{p_0}}. \end{aligned}$$

Hence, we may conclude from (2.3) that $\Vert T\Vert _{L^{p_0}\rightarrow L^{p_0,\infty }}<\infty$, finishing the proof. $\square$

#### Remark 2.3

The cancellation of the ‘bad‘ part *b* in our proofs occurs because we are able to perform our Calderón–Zygmund decomposition in the same dyadic grid as where the sparse domination occurs, see Lemma 4.6. The usual Whitney decomposition argument that is used for Calderón–Zygmund decompositions in general doubling metric measure spaces, as can be found for example in [11, 39], is not precise enough for this particular argument and we need to adapt the results so that they work with our dyadic grids.

## Proofs of the Main Results

Throughout these proofs we fix $\alpha \in \big \{0,\frac{1}{3},\frac{2}{3}\big \}^n$ and only consider cubes taken from the grid $\mathscr {D}^\alpha$. We also only consider the dyadic maximal operators $\mathscr {M}_p$ to be taken with respect to this grid to facilitate some of the arguments and for simpler constants in our estimates. Recall that $\mathcal {D}$ denotes a space of functions in $\mathbf {R}^n$ which has the property that it is dense in $L^p(w)$ for all $1\le p<\infty$ and all weights $w\in A_\infty$.

As an analogue to [32, Lemma 3.2] and [26, Lemma 6.1], our main lemma is the following:

### Lemma 3.1

Let $\mathscr {S}\subseteq \mathscr {D}^\alpha$ be $\eta$-sparse, and let $1\le p_0<p<q_0\le \infty$, $1<q<\infty$. Then there is a constant $c=c(\eta ,n,\nu )>0$ so that

$$\begin{aligned} \sum _{Q\in \mathscr {S}}\langle f\rangle _{p_0,Q}\langle g\rangle _{q_0',Q}|Q|\le c c_p(q')^{\frac{1}{p'}}\Vert f\Vert _{L^p\left( \mathscr {M}_{q(q_0/p)'}w\right) }\Vert g\Vert _{L^{p'}\left( w^{1-p'}\right) } \end{aligned}$$

for all $f,g\in \mathcal {D}$ and $w\in L^{q(q_0/p)'}_{loc}$, where $c_p$ is as in Theorem 1.3.

We point out that a similar type of result is established in [15, Theorem B].

### Remark 3.2

In the unweighted case we note that

$$\begin{aligned} \sum _{Q\in \mathscr {S}}\langle f\rangle _{p_0,Q}\langle g\rangle _{q_0',Q}|Q|\le & {} \eta ^{-1}\Vert \mathscr {M}_{p_0}f\Vert _p\Vert \mathscr {M}_{q_0'}g\Vert _{p'}\\\le & {} \eta ^{-1}\left[ \bigg (\frac{p}{p_0}\bigg )'\right] ^{\frac{1}{p_0}} \left[ \bigg (\frac{p'}{q_0'}\bigg )'\right] ^{\frac{1}{q_0'}}\Vert f\Vert _p\Vert g\Vert _{p'}. \end{aligned}$$

Thus, it appears that adding the weight accounts for the extra term $[(p_0'/p')']^{1/p_0}$ in the constant $c_p$, which depends on *p* if and only if $p_0>1$. As a matter of fact, we shall see in the proof of Lemma 3.4 that this constant appears in an application of Kolmogorov’s Lemma to the maximal operator. This extra term is what causes the additional terms in the quantitative bounds for $p_0>1$ in Theorem 1.4 and at this moment we are unsure whether it can be removed or not.

We break up the proof of the main lemma into a sequence of lemmata.

### Lemma 3.3

For all $f,g\in \mathcal {D}$ and $0<\beta \le 1$ we have

$$\begin{aligned} \sum _{Q\in \mathscr {S}}\langle f\rangle _{p_0,Q}\langle g\rangle _{q_0',Q}|Q|\lesssim \int \mathscr {M}_{p_0}\left( \left( \mathscr {M}_{q_0'} g\right) ^{1-\beta }f\right) (\mathscr {M}_{q_0'} g)^\beta \,\mathrm {d}\mu . \end{aligned}$$

### Proof

Note that for any $Q\in \mathscr {D}^\alpha$ we have

$$\begin{aligned} \langle g\rangle _{q_0',Q}=\langle g\rangle _{q_0',Q}^\beta \langle g\rangle _{q_0',Q}^{1-\beta }\le \langle g\rangle _{q_0',Q}^\beta {{\mathrm{ess\,inf}}}_{Q}\left( \mathscr {M}_{q_0'} g\right) ^{1-\beta } \end{aligned}$$

so that

$$\begin{aligned} \langle f\rangle _{p_0,Q}\langle g\rangle _{q_0',Q}\le & {} \left\langle \left( \mathscr {M}_{q_0'} g \right) ^{1-\beta }f\right\rangle _{p_0,Q}\langle g\rangle _{q_0',Q}^\beta \\\le & {} {{\mathrm{ess\,inf}}}_Q \mathscr {M}_{p_0}\left( \left( \mathscr {M}_{q_0'} g \right) ^{1-\beta }f\right) \left( \mathscr {M}_{q_0'}g\right) ^{\beta }. \end{aligned}$$

Hence, if $\mathscr {S}\subseteq \mathscr {D}^\alpha$ is sparse, we find that

$$\begin{aligned} \sum _{Q\in \mathscr {S}}\langle f\rangle _{p_0,Q}\langle g\rangle _{q_0',Q}|Q|&\lesssim \sum _{Q\in \mathscr {S}}{{\mathrm{ess\,inf}}}_Q \mathscr {M}_{p_0}\left( \left( \mathscr {M}_{q_0'} g\right) ^{1-\beta }f\right) \left( \mathscr {M}_{q_0'}g\right) ^{\beta }|E_Q|\\&\le \int \mathscr {M}_{p_0}\left( \left( \mathscr {M}_{q_0'} g\right) ^{1-\beta }f\right) \left( \mathscr {M}_{q_0'} g\right) ^ \beta \,\mathrm {d}\mu , \end{aligned}$$

as desired. $$\square $$

### Lemma 3.4

For all $1\le q<\infty$, $w\in L^q_{loc}$, $p_0<p<q_0$, and $f,g\in \mathcal {D}$, we have

$$\begin{aligned} \sum _{Q\in \mathscr {S}}\langle f\rangle _{p_0,Q}\langle g\rangle _{q_0',Q}|Q|\lesssim \tau _p\Vert f\Vert _{L^p\left( \mathscr {M}_q w\right) }\Vert \mathscr {M}_{q_0'} g\Vert _{L^{p'}\left( \left( \mathscr {M}_qw\right) ^{1-p'}\right) }, \end{aligned}$$

where

$$\begin{aligned} \tau _p=\left[ \left( \frac{p_0'}{p'}\right) '\left( \frac{p}{p_0}\right) '\right] ^{\frac{1}{p_0}}. \end{aligned}$$

For the proof of this lemma we require two results on dyadic maximal operators. By the classical result of Fefferman and Stein [17] we have

3.1 $$\begin{aligned} \Vert \mathscr {M}f\Vert _{L^{1,\infty }(w)}\le \Vert f\Vert _{L^1(\mathscr {M}w)} \end{aligned}$$

and thus $\Vert \mathscr {M}f\Vert _{L^p(w)}\le p'\Vert f\Vert _{L^p(\mathscr {M}w)}$ for $1<p<\infty$ by the Marcinkiewicz Interpolation Theorem. This implies that

3.2 $$\begin{aligned} \Vert \mathscr {M}_q f\Vert _{L^p(w)}\le \left[ \left( \frac{p}{q}\right) '\right] ^{\frac{1}{q}}\Vert f\Vert _{L^p(\mathscr {M}w)},\quad 1\le q<p<\infty . \end{aligned}$$

Moreover, as a consequence of Kolmogorov’s Lemma we have

3.3 $$\begin{aligned} \mathscr {M}((\mathscr {M}f)^\delta )\lesssim \frac{(\mathscr {M}f)^\delta }{1-\delta },\quad 0<\delta <1 \end{aligned}$$

for *f* such that $\mathscr {M}f<\infty$. For this result we refer the reader to [10, Proposition 2] or [20, Theorem 9.2.7].

### Proof

We will prove the stronger assertion

$$\begin{aligned}&\sum _{Q\in \mathscr {S}}\langle f\rangle _{p_0,Q}\langle g\rangle _{q_0',Q}|Q|\lesssim \left[ \bigg (\frac{p_0'}{r}\bigg )'\bigg (\frac{\max (r,p')'}{p_0}\bigg )'\right] ^{\frac{1}{p_0}}\\&\quad \Vert f\Vert _{L^p((\mathscr {M}w)^{(1-r)/(1-p')})}\Vert \mathscr {M}_{q_0'} g\Vert _{L^{p'}((\mathscr {M}w)^{1-r})}, \end{aligned}$$

valid for all $1<r<p_0'$, generalizing a version of the result [30, Theorem 1.7] and its proof in which the case $p_0=1$, $q_0=\infty$ is treated. The result of the lemma follows by taking $r=(p'-1)/q+1\in (1,p']$.

We set

$$\begin{aligned} \beta :=\min \left( p'\frac{r-1+p_0'-1}{p_0'(r-1)+(p_0'-1)r},1\right) \end{aligned}$$

so that $$0<\beta \le 1$$.

By Lemma 3.3 and by Hölder’s Inequality we find that

3.4 $$\begin{aligned} \begin{aligned} \sum _{Q\in \mathscr {S}}\langle f\rangle _{p_0,Q}\langle g\rangle _{q_0',Q}|Q|&\lesssim \int \mathscr {M}_{p_0}\left( \left( \mathscr {M}_{q_0'} g\right) ^{1-\beta }f\right) \left( \mathscr {M}_{q_0'} g\right) ^\beta \,\mathrm {d}\mu \\&\le I\Vert \mathscr {M}_{q_0'} g\Vert ^\beta _{L^{p'}((\mathscr {M}w)^{1-r})}, \end{aligned} \end{aligned}$$

where

$$\begin{aligned} I=\left\| \mathscr {M}_{p_0}\left( \left( \mathscr {M}_{q_0'} g \right) ^{1-\beta }f\right) \right\| _{L^{\frac{p'}{p'-\beta }}((\mathscr {M}w)^{(1-r)\beta /(\beta -p')})}. \end{aligned}$$

We will consider two cases. First assume that

$$\begin{aligned} p'\frac{r-1+p_0'-1}{p_0'(r-1)+(p'_0-1)r}\ge 1 \end{aligned}$$

and $$\beta =1$$. Then

$$\begin{aligned} (p'-1)(r-1+p_0'-1)\ge 2(p_0'-1)(r-1) \end{aligned}$$

so that

$$\begin{aligned} \frac{1-r}{1-p'}\le \frac{1}{2}\left( 1+\frac{r-1}{p_0'-1}\right) <1 \end{aligned}$$

by the assumption $r<p_0'$. Then it follows from (3.2) and (3.3) that

$$\begin{aligned} I&=\left\| \mathscr {M}_{p_0}f\right\| _{L^{p}((\mathscr {M}w)^{(1-r)/(1-p')})}\le \left[ \bigg (\frac{p}{p_0}\bigg )'\right] ^{\frac{1}{p_0}}\Vert f\Vert _{L^{p}(\mathscr {M}((\mathscr {M}w)^{(1-r)/(1-p')})))}\\&\lesssim \left( \frac{1}{1-\frac{1-r}{1-p'}}\right) ^{\frac{1}{p}}\left[ \bigg (\frac{p}{p_0}\bigg )'\right] ^{\frac{1}{p_0}}\Vert f\Vert _{L^{p}((\mathscr {M}w)^{(1-r)/(1-p')})}\\&\le \left[ 2\bigg (\frac{p_0'}{r}\bigg )'\right] ^{\frac{1}{p}}\left[ \bigg (\frac{p}{p_0}\bigg )'\right] ^{\frac{1}{p_0}}\Vert f\Vert _{L^{p}((\mathscr {M}w)^{(1-r)/(1-p')})}, \end{aligned}$$

as desired.

For the second case we assume that

$$\begin{aligned} p'\frac{r-1+p_0'-1}{p_0'(r-1)+(p_0'-1)r}<1\quad \text {and}\quad \beta =p'\frac{r-1+p_0'-1}{p_0'(r-1)+(p_0'-1)r}. \end{aligned}$$

Then, using $r<p_0'$, we note that

$$\begin{aligned} \frac{p'}{p'-\beta }=\frac{p_0+r'}{2}>p_0\quad \text {and}\quad \frac{(1-r)\beta }{\beta -p'}=\frac{1}{2}\left( 1+\frac{r-1}{p_0'-1}\right) <1. \end{aligned}$$

Hence, we may apply (3.2) and (3.3) so that

3.5 $$\begin{aligned} \begin{aligned} I&\lesssim \left( \frac{1}{1-\frac{(1-r)\beta }{\beta -p'}}\right) ^{\frac{p'-\beta }{p'}}\left( \frac{\frac{p'}{p'-\beta }}{\frac{p'}{p'-\beta }-p_0}\right) ^{\frac{1}{p_0}}\Vert (\mathscr {M}_{q_0'} g)^{1-\beta }f\Vert _{L^{\frac{p'}{p'-\beta }}\left( (\mathscr {M}w)^{(1-r)\beta /(\beta -p')}\right) }\\&\le \left[ 2\bigg (\frac{p_0'}{r}\bigg )'\right] ^{\frac{p'-\beta }{p'}}\left[ 1+2\frac{p_0}{r'}\bigg (\frac{r'}{p_0}\bigg )'\right] ^{\frac{1}{p_0}}\Vert (\mathscr {M}_{q_0'} g)^{1-\beta }f\Vert _{L^{\frac{p'}{p'-\beta }}\left( (\mathscr {M}w)^{(1-r)\beta /(\beta -p')}\right) }. \end{aligned} \end{aligned}$$

By Hölder’s Inequality we find that

$$\begin{aligned}&\Vert (\mathscr {M}_{q_0'} g )^{1-\beta }f\Vert ^{\frac{p'}{p'-\beta }}_{L^{\frac{p'}{p'-\beta }}((\mathscr {M}w)^{(1-r)\beta /(\beta -p')})}\\&\quad =\int (\mathscr {M}_{q_0'} g)^{\frac{(1-\beta )p'}{p'-\beta }}(\mathscr {M}w)^{\frac{(1-r)(\beta -1)}{\beta -p'}} |f|^{\frac{p'}{p'-\beta }}(\mathscr {M}w)^{\frac{1-r}{\beta -p'}}\,\mathrm {d}\mu \\&\quad \le \Vert \mathscr {M}_{q_0'}g\Vert _{L^{p'}((\mathscr {M}w)^{1-r})}^{\frac{p'(1-\beta )}{p'-\beta }}\Vert f\Vert ^{\frac{p'}{p'-\beta }}_{L^p((\mathscr {M}w)^{(1-r)/(1-p')})}. \end{aligned}$$

Hence, by (3.5) we have

$$\begin{aligned} I\lesssim \left[ \bigg (\frac{p_0'}{r}\bigg )'\bigg (\frac{r'}{p_0}\bigg )'\right] ^{\frac{1}{p_0}}\Vert f\Vert _{L^p((\mathscr {M}w)^{(1-r)/(1-p')})}\Vert \mathscr {M}_{q_0'} g\Vert _{L^{p'}((\mathscr {M}w)^{1-r})}^{1-\beta }. \end{aligned}$$

Thus, the result follows from (3.4).

By combining the two cases, the assertion follows. $\square$

For the proof of Lemma 3.1 we will use a result that can be found in [32, p. 8] which states that

3.6 $$\begin{aligned} \Vert \mathscr {M}f\Vert _{L^{p'}((\mathscr {M}_q w)^{1-p'})}\le \left( \frac{pq-1}{q-1}\right) ^{1-\frac{1}{pq}}\Vert f\Vert _{L^{p'}(w^{1-p'})} \end{aligned}$$

for $$1<p,q<\infty $$.

### Proof of Lemma 3.1

Setting $v:=w^{(q_0/p)'}$, it follows from (3.6) that

3.7 $$\begin{aligned} \begin{aligned} \Vert \mathscr {M}_{q_0'}g\Vert _{L^{p'}\left( \left( \mathscr {M}_{q(q_0/p)'}w\right) ^{1-p'}\right) }&=\Vert \mathscr {M}(|g|^{q_0'})\Vert ^{\frac{1}{q_0'}}_{L^{p'/q_0'}\big (\left( \mathscr {M}_q v\right) ^{1-p'/q_0'}\big )}\\&\le \left( \frac{\left( \frac{p'}{q_0'}\right) 'q-1}{q-1}\right) ^{\frac{1}{q_0'}-\frac{1}{q_0'\left( \frac{p'}{q_0'}\right) 'q}}\Vert |g|^{q_0'}\Vert ^{\frac{1}{q_0'}}_{L^{\frac{p'}{q_0'}}\big (v^{1-p'/q_0'}\big )}\\&\le \left[ \bigg (\frac{p'}{q_0'}\bigg )'\right] ^{\frac{1}{q_0'}}(q')^{\frac{1}{p'}+\frac{1}{q'}}\Vert g\Vert _{L^{p'}(w^{1-p'})}, \end{aligned} \end{aligned}$$

where we used that

$$\begin{aligned} \frac{1}{q_0'}-\frac{1}{q_0'\left( \frac{p'}{q_0'}\right) 'q}=\frac{1}{p'}+\frac{1}{q_0'\left( \frac{p'}{q_0'}\right) 'q'}\le \frac{1}{p'}+\frac{1}{q'}. \end{aligned}$$

By maximizing the function $t\mapsto t^{1/t}$ for $t\ge 1$, we note that $(q')^{1/q'}\le e^{1/e}$. Hence, by combining Lemma 3.4 and (3.7), the result follows. $\square$

### Proof of Theorem 1.3

Set $v:=w^{(q_0/p)'}\in A_1$ and let $\kappa$ be the constant from Proposition 2.1(iii). Setting $q=1+1/(\kappa [v]_{A_\infty })$, we have $q'\simeq [v]_{A_\infty }$ and

$$\begin{aligned} \mathscr {M}_{q(q_0/p)'}w=(\mathscr {M}_q v)^{\frac{1}{(q_0/p)'}}\lesssim [v]^{\frac{1}{(q_0/p)'}}_{A_1}w. \end{aligned}$$

Hence, from Lemma 3.1 it follows that

$$\begin{aligned} |\langle Tf,g\rangle |\lesssim c_p[v]^{\frac{1}{p'}}_{A_\infty }[v]^{^{\frac{1}{p(q_0/p)'}}}_{A_1}\Vert f\Vert _{L^p(w)}\Vert g\Vert _{L^{p'}(w^{1-p'})}, \end{aligned}$$

proving the result. $\square$

### Proof of Theorem 1.4

The proof uses arguments similar to the ones presented in the proof of Proposition 2.2. We use the equivalence

3.8 $$\begin{aligned}&\Vert T\Vert _{L^{p_0}(w)\rightarrow L^{p_0,\infty }(w)}\nonumber \\&\quad \simeq \sup _{\Vert f\Vert _{L^{p_0}(w)}=1}\sup _{\begin{array}{c} E\subseteq \mathbf {R}^n\\ 0<w(E)<\infty \end{array}}\inf _{\begin{array}{c} E'\subseteq E\\ w(E)\le 2 w(E') \end{array}}\sup _{|h|\le \chi _{E'}} w(E)^{\frac{1}{p_0}-1}\left| \int (Tf)hw\,\mathrm {d}\mu \right| . \end{aligned}$$

Fixing a function $f\in \mathcal {D}$ with $\Vert f\Vert _{L^{p_0}(w)}=1$ and a measurable set *E*, we set $\Omega :=\{\mathscr {M}^{\mathscr {B}}(|f|^{p_0})> 2c[w]_{A_1} w(E)^{-1}\}$, where $\mathscr {M}^{\mathscr {B}}$ denotes the uncentred maximal operator with respect to all balls $B\subseteq \mathbf {R}^n$ and where $c=c(n,\nu )>0$ is the constant appearing in the inequality $\Vert \mathscr {M}^{\mathscr {B}}\phi \Vert _{L^{1,\infty }(w)}\le c[w]_{A_1}\Vert \phi \Vert _{L^1(w)}$, which is a consequence of (3.1). We have

3.9 $$\begin{aligned} w(\Omega )\le \frac{c[w]_{A_1}w(E)}{2c[w]_{A_1}}\int \!|f|^{p_0}w\,\mathrm {d}\mu =\frac{w(E)}{2} \end{aligned}$$

and thus, setting $E':=E\backslash \Omega$, we have $w(E')\ge w(E)-w(\Omega )\ge w(E)/2$.

By applying Lemma 4.6 with $|f|^{p_0}\in L^1$, we obtain a disjoint collection $\mathscr {P}\subseteq \mathscr {D}^\alpha$ of cubes so that $\Omega =\cup _{P\in \mathscr {P}}P$ and functions *g*, *b* so that $|f|^{p_0}=g+b$, where

$$\begin{aligned} g=|f|^{p_0}\chi _{\Omega ^c}+\sum _{P\in \mathscr {P}}\langle |f|^{p_0}\rangle _{1,P}\chi _P \end{aligned}$$

and

$$\begin{aligned} \Vert g\Vert _\infty \lesssim \frac{[w]_{A_1}}{w(E)}. \end{aligned}$$

Picking a function *h* satisfying $|h|\le \chi _{E'}$ and $hw\in \mathcal {D}$, we apply the sparse domination property to the pair *f*, *hw* to find a sparse collection $\mathscr {S}\subseteq \mathscr {D}^\alpha$ so that, by using Lemma 3.1 with the weight $w\chi _{E'}$, for all $p_0<p<q_0$ and $1<q<\infty$ we have

3.10 $$\begin{aligned} \begin{aligned} \left| \int (Tf)hw\,\mathrm {d}\mu \right|&=|\langle Tf,hw\rangle |\le \sum _{\begin{array}{c} Q\in \mathscr {S}\\ Q\cap E'\ne \emptyset \end{array}}\left( \frac{1}{|Q|}\int _Q\!g\,\mathrm {d}\mu \right) ^{\frac{1}{p_0}}\langle hw\rangle _{q_0',Q}|Q|\\&\lesssim c_p(q')^{\frac{1}{p'}}\Vert |g|^{\frac{1}{p_0}}\Vert _{L^p \left( \mathscr {M}_{q(q_0/p)'}(w\chi _{E'})\right) }\Vert hw\Vert _{L^{p'}(w^{1-p'})}\\&\lesssim c_p(q')^{\frac{1}{p'}}[w]_{A_1} ^{\frac{1}{p_0}-\frac{1}{p}}w(E)^{\frac{1}{p}-\frac{1}{p_0}}\Vert g\Vert ^{\frac{1}{p}} _{L^1\left( \mathscr {M}_{q(q_0/p)'}(w\chi _{E'})\right) }w(E')^{\frac{1}{p'}}. \end{aligned} \end{aligned}$$

Note here that we have used the fact that the terms involving *b* cancel in the exact same way as they do in the proof of Proposition 2.2.

Similar to what is done in [26, 32, 37], we deal with the term involving *g* as follows: We remark that for a cube $P\in \mathscr {D}^\alpha$ we have

3.11 $$\begin{aligned} \mathscr {M}(\phi \chi _{P^c})(x)={{\mathrm{ess\,inf}}}_P\mathscr {M}(\phi \chi _{P^c})\quad \text {for all } x\in P. \end{aligned}$$

Indeed, let $x,y\in P$ and let $R\in \mathscr {D}^\alpha$ so that $x\in R$. Then either $R\subseteq P$ or $P\subseteq R$. In the first case we have $\langle \phi \chi _{P^c}\rangle _{1,R}=0$, while in the second case we have $y\in R$ and thus $\langle \phi \chi _{P^c}\rangle _{1,R}\le \mathscr {M}(\phi \chi _{P^c})(y)$. Thus, we may conclude that $\mathscr {M}(\phi \chi _{P^c})(x)\le \mathscr {M}(\phi \chi _{P^c})(y)$, proving (3.11) by symmetry. Using this result, we find, since $E'\subseteq P^c$ for all $P\in \mathscr {P}$, that

$$\begin{aligned} \int _\Omega \!|g|\mathscr {M}_{q(q_0/p)'}(w\chi _{E'})\,\mathrm {d}\mu&\le \sum _{P\in \mathscr {P}}{{\mathrm{ess\,inf}}}_P\mathscr {M}_{q(q_0/p)'}(w\chi _{P^c})\int _P\!|f|^{p_0}\,\mathrm {d}\mu \\&\le \int _\Omega \!|f|^{p_0}\mathscr {M}_{q(q_0/p)'}w\,\mathrm {d}\mu . \end{aligned}$$

Since $g=|f|^{p_0}$ on $\Omega ^c$, we conclude that

3.12 $$\begin{aligned} \Vert g\Vert _{L^1\left( \mathscr {M}_{q(q_0/p)'}(w\chi _{E'})\right) }\le \Vert f\Vert ^{p_0}_{L^{p_0}(\mathscr {M}_{q(q_0/p)'}w)}. \end{aligned}$$

We first assume that $q_0<\infty$. We set $v:=w^{(q_0/p_0)'}\in A_1$ and choose

$$\begin{aligned} p=p_0+\frac{q_0-p_0}{1+2\kappa [v]_{A_\infty }},\quad q=\frac{2+2\kappa [v]_{A_\infty }}{1+2\kappa [v]_{A_\infty }}. \end{aligned}$$

Then we have $q'=2+2\kappa [v]_{A_\infty }\simeq [v]_{A_\infty }$, and

$$\begin{aligned} q\bigg (\frac{q_0}{p}\bigg )'\bigg /\bigg (\frac{q_0}{p_0}\bigg )'=1+\frac{1}{\kappa [v]_{A_\infty }} \end{aligned}$$

so that it follows from Proposition 2.1(iii) that

$$\begin{aligned} \mathscr {M}_{q(q_0/p)'}w\lesssim [v]_{A_1}^{\frac{1}{(q_0/p_0)'}}w. \end{aligned}$$

Thus, it follows from (3.10), (3.12), and Proposition 2.1(ii) that

3.13 $$\begin{aligned} w(E)^{\frac{1}{p_0}-1}\left| \int (Tf)hw\,\mathrm {d}\mu \right|\lesssim & {} c_p[v]^{\frac{1}{p'}}_{A_\infty }[w]^{\frac{1}{p_0}-\frac{1}{p}}_{A_1}[v]^{\frac{1}{p(q_0/p_0)'}}_{A_1}\nonumber \\\le & {} c_p[v]^{\frac{1}{p'}}_{A_\infty }\left( [w]_{A_1}[w]_{{{\mathrm{RH}}}_{(q_0/p_0)'}}\right) ^{\frac{1}{p_0}}. \end{aligned}$$

Next, we note that

$$\begin{aligned} \frac{1}{p'}=\frac{q_0-1+2\kappa [v]_{A_\infty }(p_0-1)}{q_0+2\kappa [v]_{A_\infty }p_0}\le \frac{q_0-1}{2\kappa [v]_{A_\infty }p_0}+\frac{1}{p_0'} \end{aligned}$$

and thus

$$\begin{aligned}{}[v]_{A_\infty }^{\frac{1}{p'}}\lesssim [v]_{A_\infty }^{\frac{1}{p_0'}}. \end{aligned}$$

Moreover, we compute

$$\begin{aligned} c_p&=\left[ \frac{2(q_0-1)(q_0+2\kappa [v]_{A_\infty }p_0)}{2\kappa [v]_{A_\infty }(q_0-p_0)}\right] ^{\frac{1}{q_0'}}\\&\quad \times \,\left[ \frac{p_0(q_0+2\kappa [v]_{A_\infty })(q_0-1+2\kappa [v]_{A_\infty }(p_0-1))}{(q_0-p_0)^2}\right] ^{\frac{1}{p_0}}\\&\lesssim [v]_{A_\infty }^{\frac{1}{p_0}}\left[ 1+(p_0-1)[v]_{A_\infty }\right] ^{\frac{1}{p_0}}. \end{aligned}$$

Hence, it follows from (3.8) and (3.13) that

$$\begin{aligned} \Vert T\Vert _{L^{p_0}(w)\rightarrow L^{p_0,\infty }(w)}\lesssim [v]_{A_\infty }\left[ 1+(p_0-1)[v]_{A_\infty }\right] ^{\frac{1}{p_0}}\left( [w]_{A_1}[w]_{{{\mathrm{RH}}}_{(q_0/p_0)'}}\right) ^{\frac{1}{p_0}}. \end{aligned}$$

The result follows by considering the cases $p_0=1$ and $p_0>1$ separately.

Now we assume that $q_0=\infty$. Taking $q=1+1/(\kappa [w]_{A_\infty })$ we have $q'\simeq [w]_{A_\infty }$. Thus, from (3.10) and Proposition 2.1(iii) we obtain

3.14 $$\begin{aligned} w(E)^{\frac{1}{p_0}-1}\left| \int (Tf)hw\,\mathrm {d}\mu \right| \lesssim c_p[w]^{\frac{1}{p'}}_{A_\infty }[w]^{\frac{1}{p_0}}_{A_1} \end{aligned}$$

for all $p_0<p<\infty$. Choosing $p=p_0+1/(\log (e+[w]_{A_\infty }))$, we have

$$\begin{aligned} \frac{1}{p'}=\frac{1+(p_0-1)\log (e+[w]_{A_\infty })}{1+p_0\log (e+[w]_{A_\infty })}\le \frac{1}{p_0\log (e+[w]_{A_\infty })}+\frac{1}{p_0'} \end{aligned}$$

so that

3.15 $$\begin{aligned}{}[w]_{A_\infty }^{\frac{1}{p'}}\lesssim [w]_{A_\infty }^{\frac{1}{p_0'}}. \end{aligned}$$

Moreover, we compute

$$\begin{aligned} c_p&=p\left[ p_0(1+(p_0-1)\log (e+[w]_{A_\infty }))(1+p_0\log (e+[w]_{A_\infty }))\right] ^{\frac{1}{p_0}}\\&\lesssim \big [1+(p_0-1)\log (e+[w]_{A_\infty })\big ]^{\frac{1}{p_0}}\log (e+[w]_{A_\infty })^{\frac{1}{p_0}}. \end{aligned}$$

Hence, by (3.8), (3.14), and (3.15), we conclude that

$$\begin{aligned}&\Vert T\Vert _{L^{p_0}(w)\rightarrow L^{p_0,\infty }(w)}\\&\quad \lesssim [w]^{\frac{1}{p_0}}_{A_1}[w]^{\frac{1}{p_0'}}_{A_\infty }\big [1+(p_0-1)\log (e+[w]_{A_\infty })\big ]^{\frac{1}{p_0}}\log (e+[w]_{A_\infty })^{\frac{1}{p_0}}. \end{aligned}$$

By considering the cases $p_0=1$ and $p_0>1$ separately, the desired result follows. $\square$

### Proof of Theorem 1.5

We use the equivalence

3.16 $$\begin{aligned} \left\| \frac{T^*f}{w^{\frac{1}{q_0'}}}\right\| _{L^{q_0',\infty }(w)}\simeq \sup _{\begin{array}{c} E\subseteq \mathbf {R}^n\\ 0<w(E)<\infty \end{array}}\inf _{\begin{array}{c} E'\subseteq E\\ w(E)\le 2 w(E') \end{array}}\sup _{|h|\le \chi _{E'}} w(E)^{-\frac{1}{q_0}}\left| \left\langle \frac{T^*f}{w^{\frac{1}{q_0'}}},hw\right\rangle \right| . \end{aligned}$$

Let $f\in \mathcal {D}$ with $\Vert f\Vert _{q_0'}=1$ and let $E\subseteq \mathbf {R}^n$ with $0<w(E)<\infty$. We denote by $\mathscr {M}_w^{\mathscr {B}}$ the uncentred maximal operator over balls with respect to the measure $w\,\mathrm {d}\mu$. Then we define

$$\begin{aligned} \Omega :=\Big \{\mathscr {M}_w^{\mathscr {B}}\Big (\frac{|f|^{q_0'}}{w}\Big )> 2cw(E)^{-1}\Big \}, \end{aligned}$$

where $c=c(n,\nu )>0$ is the constant appearing in the inequality $\Vert \mathscr {M}_w^{\mathscr {B}}\phi \Vert _{L^{1,\infty }(w)}\le c\Vert \phi \Vert _{L^1(w)}$. We have

$$\begin{aligned} w(\Omega )\le \frac{cw(E)}{2c}\int \!\frac{|f|^{q_0'}}{w}w\,\mathrm {d}\mu =\frac{w(E)}{2} \end{aligned}$$

which, setting $E':=E\backslash \Omega$, implies that $w(E')\ge w(E)-w(\Omega )\ge w(E)/2$.

By applying the Whitney Decomposition Theorem to $\Omega$, see Theorem 4.7, we obtain a disjoint collection $\mathscr {P}\subseteq \mathscr {D}^\alpha$ of cubes so that $\Omega =\cup _{P\in \mathscr {P}}P$ with the property that for each $P\in \mathscr {P}$ there exists a ball *B*(*P*) containing *P* so that $B(P)\cap \Omega ^c\ne \emptyset$ and $|B(P)|\lesssim |P|$, where the implicit constant depends only on *n* and $\nu$, see also the proof of Lemma 4.6. Moreover, we obtain functions *g*, *b* so that $|f|^{q_0'}=g+b$, where

$$\begin{aligned} g=|f|^{q_0'}\chi _{\Omega ^c}+\sum _{P\in \mathscr {P}}\langle |f|^{q_0'}\rangle _{1,P}\chi _P. \end{aligned}$$

Next, we pick a function *h* satisfying $|h|\le \chi _{E'}$ and $hw^{1/q_0}\in \mathcal {D}$, and fix a $p_0<p<q_0$ to be chosen later. We apply the sparse domination property to the pair $hw^{1/q_0}$, *f* to find a sparse collection $\mathscr {S}\subseteq \mathscr {D}^\alpha$ so that, by applying Lemma 3.1 with the weight $w^{1/(q_0/p)'}$, we find that for all $1<q<\infty$ we have

3.17 $$\begin{aligned} \begin{aligned} \left| \left\langle \frac{T^*f}{w^{\frac{1}{q_0'}}},hw\right\rangle \right|&=\left| \left\langle f,T\left( hw^{\frac{1}{q_0}}\right) \right\rangle \right| \le \sum _{\begin{array}{c} Q\in \mathscr {S}\\ Q\cap E'\ne \emptyset \end{array}}\left\langle hw^{\frac{1}{q_0}}\right\rangle _{p_0,Q}\left( \frac{1}{|Q|}\int _Q\!g\,\mathrm {d}\mu \right) ^{\frac{1}{q_0'}}|Q|\\&\lesssim c_p(q')^{\frac{1}{p'}}\Vert hw^{\frac{1}{q_0}}\Vert _{L^{p} \left( (\mathscr {M}_{q}w)^{\frac{1}{(q_0/p)'}}\right) }\Vert |g| ^{\frac{1}{q'_0}}\Vert _{L^{p'}\left( w^{\frac{1-p'}{(q_0/p)'}}\right) }\\&=c_p(q')^{\frac{1}{p'}}\Vert h w^{\frac{1}{q_0}}\Vert _{L^{p}\left( (\mathscr {M}_{q}w) ^{\frac{1}{(q_0/p)'}}\right) }\\&\quad \times \, \left( \int _{\Omega ^c}\!|f|^{p'}w^{\frac{1-p'}{(q_0/p)'}}\, \mathrm {d}\mu +\sum _{P\in \mathscr {P}}\langle f\rangle _{q_0',P}^{p'} \int _P\!w^{\frac{1-p'}{(q_0/p)'}}\,\mathrm {d}\mu \right) ^{\frac{1}{p'}}, \end{aligned} \end{aligned}$$

where the terms involving *b* cancel in the same way as before.

Choosing $q=1+1/(\kappa [w]_{A_\infty })$ so that $q'\simeq [w]_{A_\infty }$, it follows from Proposition 2.1(iii) that

3.18 $$\begin{aligned} (q')^{\frac{1}{p'}}\Vert h w^{\frac{1}{q_0}}\Vert _{L^{p}((\mathscr {M}_{q}w)^{\frac{1}{(q_0/p)'}})}\lesssim & {} [w]^{\frac{1}{p'}}_{A_\infty }[w]_{A_1}^{\frac{1}{p(q_0/p)'}}\left( \int |h|^p w^{\frac{p}{q_0}}w^{\frac{1}{(q_0/p)'}}\,\mathrm {d}\mu \right) ^{\frac{1}{p}}\nonumber \\\le & {} [w]^{\frac{1}{p'}}_{A_\infty }[w]_{A_1}^{\frac{1}{p(q_0/p)'}}w(E')^{\frac{1}{p}}. \end{aligned}$$

Next, since $|f|\lesssim w(E)^{-1/q_0'}w^{1/q_0'}$ in $\Omega ^c$, we have

3.19 $$\begin{aligned} \int _{\Omega ^c}\!|f|^{p'}w^{\frac{1-p'}{(q_0/p)'}}\,\mathrm {d}\mu \le w(E)^{\frac{q_0'-p'}{q_0'}}\int _{\Omega ^c}\!|f|^{q_0'}w^{\frac{p'-q_0'}{q_0'}}w^{\frac{q_0'-p'}{q_0'}}\,\mathrm {d}\mu \le w(E)^{\frac{q_0'-p'}{q_0'}}. \end{aligned}$$

Furthermore, fixing a $P\in \mathscr {P}$ and $x\in B(P)\cap \Omega ^c$, we have

$$\begin{aligned} \langle f\rangle _{q_0',P}^{p'-q_0'}&\lesssim \langle f\rangle _{q_0',B(P)}^{p'-q_0'}\le \left[ \mathscr {M}^{\mathscr {B}}_w\Big (\frac{|f|^{q_0'}}{w}\Big )(x)\right] ^{\frac{p'-q_0'}{q_0'}}\langle w\rangle _{1,B(P)}^{\frac{p'-q_0'}{q_0'}}\\&\lesssim w(E)^{\frac{q_0'-p'}{q_0'}}\langle w\rangle _{1,B(P)}^{\frac{p'-q_0'}{q_0'}} \end{aligned}$$

and

$$\begin{aligned} \Big \langle w^{\frac{q_0'-p'}{q_0'}}\Big \rangle _{1,P}\lesssim & {} [w]_{A_1}^{\frac{p'-q_0'}{q_0'}}\Big \langle (\mathscr {M}^{\mathscr {B}}w)^{\frac{q_0'-p'}{q_0'}}\Big \rangle _{1,B(P)}\\\le & {} [w]_{A_1}^{\frac{p'-q_0'}{q_0'}}\langle w\rangle _{1,B(P)}^{\frac{q_0'-p'}{q_0'}} \end{aligned}$$

so that

3.20 $$\begin{aligned} \sum _{P\in \mathscr {P}}\langle f\rangle _{q_0',P}^{p'}\int _P\!w^{\frac{1-p'}{(q_0/p)'}}\,\mathrm {d}\mu\lesssim & {} [w]^{\frac{p'-q_0'}{q_0'}}_{A_1}w(E)^{\frac{q_0'-p'}{q_0'}}\sum _{P\in \mathscr {P}}\langle |f|^{q_0'}\rangle _{1,P}|P|\nonumber \\\le & {} [w]^{\frac{p'-q_0'}{q_0'}}_{A_1}w(E)^{\frac{q_0'-p'}{q_0'}}. \end{aligned}$$

Thus, by combining (3.18),(3.19), and (3.20) with (3.17), we conclude that

3.21 $$\begin{aligned} \begin{aligned} \left| \left\langle \frac{T^*f}{w^{\frac{1}{q_0'}}},hw\right\rangle \right|&\lesssim c_p[w]_{A_\infty }^{\frac{1}{p'}}[w]_{A_1}^{\frac{1}{p(q_0/p)'}}[w]_{A_1}^{\frac{p'-q_0'}{p'q_0'}}w(E')^{\frac{1}{p}}w(E)^{\frac{q_0'-p'}{p'q_0'}}\\&\le c_p[w]_{A_\infty }^{\frac{1}{q_0'}}[w]_{A_1}^{\frac{2}{p(q_0/p)'}}w(E)^{\frac{1}{q_0}}. \end{aligned} \end{aligned}$$

By writing $L:=\log (e+[w]_{A_1})$ and choosing

$$\begin{aligned} p=p_0\frac{q_0}{q_0+L}+q_0\frac{L}{q_0+L}\in (p_0,q_0) \end{aligned}$$

we have

$$\begin{aligned}{}[w]_{A_1}^{\frac{2}{p(q_0/p)'}}=[w]_{A_1}^{\frac{2}{(q_0/p_0)'(p_0+L)}}\le e^{2/e} \end{aligned}$$

and

$$\begin{aligned} c_p= & {} \left[ \frac{q_0-1}{q_0-p_0}(p_0+L)\right] ^ {\frac{1}{q_0'}} \left[ p_0\bigg (\frac{q_0}{p_0}\bigg )' \frac{p_0+L}{L}\frac{(p_0-1)\left( \frac{q_0}{p_0}\right) ' +\frac{q_0-1}{q_0-p_0}L}{L}\right] ^{\frac{1}{p_0}}\\\lesssim & {} L^{\frac{1}{q_0'}}. \end{aligned}$$

Thus, by (3.16) and (3.21) we have

$$\begin{aligned} \left\| \frac{T^*f}{w^{\frac{1}{q_0'}}}\right\| _{L^{q_0',\infty }(w)}\lesssim \left( [w]_{A_\infty }L\right) ^{\frac{1}{q_0'}}, \end{aligned}$$

as desired. $$\square $$

## Extensions of the Results to Spaces of Homogeneous Type

This section is dedicated to extending our main results to spaces of homogeneous type $(X,d,\mu )$. Here *X* is a set equipped with a quasimetric *d*, i.e. a mapping satisfying the usual properties of a metric except for the triangle inequality, which is replaced by the estimate

$$\begin{aligned} d(x,y)\le A(d(x,z)+d(z,y)) \end{aligned}$$

for a constant $A\ge 1$, and $\mu$ is a Borel measure on *X* satisfying the doubling property, i.e. there is a $C>0$ such that

$$\begin{aligned} \mu (B(x;2r))\le C\mu (B(x;r)) \end{aligned}$$

for all $x\in X$, $r>0$. Taking the smallest such *C* we set $\nu :=\log _2 C$. Furthermore, we write $|E|:=\mu (E)$ for all Borel sets $E\subseteq X$. The doubling property implies that for $x\in X$ and $R\ge r>0$ we have

4.1 $$\begin{aligned} |B(x;R)|\le C\left( \frac{R}{r}\right) ^\nu |B(x;r)|. \end{aligned}$$

In turn, this implies that if $y\in B(x;R)$ for $x\in X$, then for $0<r\le 2AR$ we have

4.2 $$\begin{aligned} |B(x;R)|\le C\left( \frac{2AR}{r}\right) ^\nu |B(y;r)|. \end{aligned}$$

We make the additional assumption that $0<|B|<\infty$ for all balls $B\subseteq X$. This property ensures that *X* is separable [7, Proposition 1.6].

Finally, we make the assumption that Lebesgue’s Differentiation Theorem holds. This holds, for example, when *X* is a domain in $\mathbf {R}^n$. Indeed, more generally, if $A=1$ (that is, (*X*, *d*) is a metric space) and $\mu$ is an inner regular Borel outer measure, then Lebesgue’s Differentiation Theorem holds, see [22, Sect. 14]. This assumption is used for the $L^\infty$ bound on the good part in our Calderón–Zygmund decompositions.

We will consider the situations where *X* is unbounded and where *X* is bounded separately, the latter situation being simpler. To facilitate this, we impose that the underlying quasimetric space (*X*, *d*) has exactly one of the following properties:

(I) There is a constant $\gamma >0$ so that
  4.3 $$\begin{aligned} {{\mathrm{diam}}}(B(x;r))\ge \gamma r \end{aligned}$$
  for all $$x\in X$$, $$r>0$$;
(II) $${{\mathrm{diam}}}X<\infty $$.

We note that property (I) and property (II) are mutually exclusive, since (I) implies that *X* is unbounded. The extra assumption for the unbounded case is not too restrictive in the sense that the unbounded spaces in our applications usually do satisfy property (I). We point out that when (*X*, *d*) is a connected metric space, then it satisfies either (I) or (II):

### Proposition 4.1

Suppose *X* is metric, connected, and unbounded. Then (I) holds with $\gamma =1$.

### Proof

Let $r>\varepsilon >0$. The assumptions on *X* imply that $X\ne \overline{B(x;r-\varepsilon )}\cup B(x;r)^c$ and thus we can pick $y\in B(x;r)\backslash \overline{B(x;r-\varepsilon )}$ so that ${{\mathrm{diam}}}(B(x;r))\ge d(x,y)\ge r-\varepsilon$, proving the result. $\square$

A non-connected example where (I) holds with $\gamma =1/2$ is the subset $(-\infty ,0)\cup (1,2)$ of the real line. An example where (I) fails is any metric space that has an isolated point.

We will use the following definition of a dyadic system in *X*.

### Definition 4.2

Let $0<c_0\le C_0<\infty$ and $0<\delta <1$. If for each $k\in \mathbf {Z}$ we have a pairwise disjoint collection $\mathscr {D}_k=(Q^k_j)_{j\in J_k}$ of measurable subsets of *X* and a collection of points $(z^k_j)_{j\in J_k}$, then we call $(\mathscr {D}_k)_{k\in \mathbf {Z}}$ a *dyadic system* in *X* with parameters $c_0$, $C_0$, $\delta$, if it satisfies the following properties:

(i) for all $k\in \mathbf {Z}$ we have
  $$\begin{aligned} X=\bigcup _{j\in J_k}Q_j^k; \end{aligned}$$
(ii) for $l\ge k$, if $Q\in \mathscr {D}_l$ and $Q'\in \mathscr {D}_k$, we have that either $Q\cap Q'=\emptyset$ or $Q\subseteq Q'$;
(iii) for each $k\in \mathbf {Z}$ and $j\in J_k$ we have
  $$\begin{aligned} B(z^k_j;c_0\delta ^k)\subseteq Q^k_j\subseteq B(z^k_j;C_0\delta ^k); \end{aligned}$$
(iv) for $l\ge k$, if $Q^l_{j'}\subseteq Q^k_j$, then $B(z^l_{j'};C_0\delta ^k)\subseteq B(z^k_j;C_0\delta ^k)$.

The elements of a dyadic system are called cubes. We call $z^k_j$ the *centre* of $Q^k_j$. If $Q\in \mathscr {D}_k$, then we call the unique cube $Q'\in \mathscr {D}_{k-1}$ so that $Q\subseteq Q'$, the *parent* of *Q*. Furthermore, we say that *Q* is a *child* of $Q'$. Note that it is possible that for a cube *Q* there exists more than one $k\in \mathbf {Z}$ so that $Q\in \mathscr {D}_k$. Hence, when speaking of a child or the parent of *Q*, this should be with respect to a specific $k\in \mathbf {Z}$ where $Q\in \mathscr {D}_k$ to avoid ambiguity.

For a detailed discussion on the construction of dyadic systems and for the following theorem we refer the reader to [25] and references therein.

### Theorem 4.3

There exist $0<c_0<C_0<\infty$, $0<\delta <1$, $\rho >0$ and a positive integer *K*, so that there are dyadic system $\mathscr {D}^1,\ldots ,\mathscr {D}^K$ in *X* with parameters $c_0$, $C_0$, $\delta$ so that for each $x\in X$ and $r>0$ there exists an $\alpha \in \{1,\ldots , K\}$ and $Q\in \mathscr {D}^\alpha$ so that

$$\begin{aligned} B(x;r)\subseteq Q\quad \text {and}\quad {{\mathrm{diam}}}(Q)\le \rho r. \end{aligned}$$

Writing $\mathscr {D}:=\cup _{\alpha =1}^K\mathscr {D}^\alpha$, one defines the respective notions for weight classes accordingly. Likewise, we say that a collection $\mathscr {S}\subseteq \mathscr {D}$ is called $\eta$-*sparse* for $0<\eta \le 1$ if for each $\alpha \in \big \{1,\ldots , K\big \}$ there is a pairwise disjoint collection $(E_Q)_{Q\in \mathscr {S}\cap \mathscr {D}^\alpha }$ of measurable sets so that $E_Q\subseteq Q$ and $|Q|\le \eta ^{-1}|E_Q|$.

For our main results we require that the Calderón–Zygmund decompositions we take are adapted to the dyadic grids obtained from this theorem. The standard Calderón–Zygmund decomposition as found in [11] is not precise enough for these purposes, see also Remark 2.3.

For $1\le p_0<q_0\le \infty$ we may define the class $S(p_0,q_0)$ as the class of those operators *T* that satisfy the property that there is a constant $c>0$ and an $0<\eta \le 1$ so that for each pair of functions *f*, *g* in an appropriately large class of functions on *X* there is an $\eta$-sparse collection $\mathscr {S}\subseteq \mathscr {D}$ so that

$$\begin{aligned} |\langle Tf,g\rangle |\le c\sum _{Q\in \mathscr {S}}\langle f\rangle _{p_0,Q}\langle g\rangle _{q_0',Q}|Q|. \end{aligned}$$

The remainder of this section will be dedicated to proving the following result:

### Theorem 4.4

Let $1\le p_0<q_0\le \infty$ and suppose that (*X*, *d*) satisfies either property (I) or property (II). Then for $T\in S(p_0,q_0)$, the results of Theorems 1.3 and 1.4 remain true, where the dependence on *n* of the constants changes to dependence on the parameters of the dyadic system (and also $\gamma$ in the case (I)). Similarly, the results of Theorem 1.5 remain true in the case that property (I) is satisfied.

The main difficulty arises when one wants to take Calderón–Zygmund decompositions. We remark that in the cases (I) and (II) one can use the standard maximal cube arguments and localization arguments, respectively, to conclude that our dyadic maximal operators satisfy the usual weak and strong boundedness results. The Lemmata in Sect. 3 all follow in the more general setting in the same way as they have been presented, where we replace the set of test functions $\mathcal {D}$ by another appropriate class of functions that is dense in $L^p(w)$ for all $1\le p<\infty$, $w\in A_\infty$ such as the linear span of the indicator functions functions over the balls in *X*.

From now on we consider a fixed dyadic system $\mathscr {D}^*=\cup _{k\in \mathbf {Z}}\mathscr {D}_k$ in *X* with parameters $c_0$, $C_0$, $\delta$.

We first assume that we are in the easier case (II). We define the maximal operator $\mathscr {M}$ with respect to the cubes $Q\in \mathscr {D}^*$ by $\mathscr {M}f:=\sup _{Q\in \mathscr {D}^*}\langle f\rangle _{1,Q}\chi _Q$.

### Lemma 4.5

(Calderón–Zygmund Lemma in the case (II)) Let $f\in L^1$, $\lambda >0$, and let $\Omega :=\{\mathscr {M}f>\lambda \}$. If $\Omega \ne X$, then we can find a pairwise disjoint collection of cubes $\mathscr {P}\subseteq \mathscr {D}^*$ and a constant $c>0$, depending only on the parameters of the dyadic system, the doubling dimension $\nu$, and the quasimetric constant *A*, so that

$$\begin{aligned} \Omega =\bigcup _{P\in \mathscr {P}}P, \end{aligned}$$

and for each $P\in \mathscr {P}$

$$\begin{aligned} \lambda <\langle f\rangle _{1,P}\le c\lambda . \end{aligned}$$

### Proof

Fix $k_0\in \mathbf {Z}$ small enough so that $c_0\delta ^{k_0}>{{\mathrm{diam}}}X$. Then for any $x\in X$ we have $B(x;c_0\delta ^{k_0})=X$. Hence, it follows from property (iii) of dyadic systems that $\mathscr {D}_{k_0}=\{X\}$.

Note that $\Omega \ne X$ implies that $\langle f\rangle _{1,X}\le \lambda$. Let $x\in \Omega$. Then the set

$$\begin{aligned} K_x:=\{k>k_0\mid \text {there is a } Q\in \mathscr {D}_k,\, x\in Q,\, \langle f\rangle _{1,Q}>\lambda \} \end{aligned}$$

is non-empty. Thus, by well-orderedness there is a minimal $k_x\in K_x$, and thus a cube $P_x\in \mathscr {D}_{k_x}$ that contains *x* so that $\langle f\rangle _{1,P_x}>\lambda$. By minimality of $k_x$, it follows that $\langle f\rangle _{1,p(P_x)}\le \lambda$, where $p(P_x)\in \mathscr {D}_{k_x-1}$ denotes the parent of $P_x$. By (4.2) and property (iii) of dyadic systems this implies that

$$\begin{aligned} \lambda <\langle f\rangle _{1,P_x}\le c\langle f\rangle _{1,p(P_x)}\le c\lambda , \end{aligned}$$

with $$c=C(2AC_0/(c_0\delta ))^\nu $$.

It remains to show that the hereby obtained collection $\mathcal {P}=(P_x)_{x\in X}$ is pairwise disjoint. Indeed, assume that $P_1,P_2\in \mathcal {P}$ so that $P_1\cap P_2\ne \emptyset$. We have either $P_1\subseteq P_2$ or $P_2\subseteq P_1$ by property (ii) of dyadic systems. Without loss of generality we assume the first. Pick $x\in X$ so that $P_1=P_x$. Since $x\in P_2$ and $\langle f\rangle _{1,P_2}>\lambda$, minimality of $k_x$ implies that $P_2\in \mathscr {D}_l$ for some $l\ge k_x$. Again by property (ii) of dyadic systems, this implies that $P_2\subseteq P_1$, proving that $P_1=P_2$. The assertion follows. $\square$

Next, we consider the case (I). We define the maximal operator $\mathscr {M}^{\mathscr {B}}$ with respect to the balls $B\subseteq X$ by $\mathscr {M}^{\mathscr {B}} f:=\sup _{B}\langle f\rangle _{1,B}\chi _B$.

### Lemma 4.6

(Calderón–Zygmund Lemma in the case (I)) Let $f\in L^1$, $\lambda >0$, and let $\Omega :=\{\mathscr {M}^{\mathscr {B}} f>\lambda \}$. If $\Omega \ne X$, then we can find a pairwise disjoint collection of cubes $\mathscr {P}\subseteq \mathscr {D}^*$ and a constant $c>0$, depending only on the parameters of the dyadic system, the doubling dimension $\nu$, the quasimetric constant *A*, and $\gamma$, so that

$$\begin{aligned} \Omega =\bigcup _{P\in \mathscr {P}}P, \end{aligned}$$

and for each $P\in \mathscr {P}$

$$\begin{aligned} \langle f\rangle _{1,P}\le c\lambda . \end{aligned}$$

For the proof we use a version of the Whitney Decomposition Theorem. Note that the diameter assumption (4.3) together with property (iii) of dyadic systems implies that for any $Q\in \mathscr {D}_k$ we have

4.4 $$\begin{aligned} \gamma c_0\delta ^k\le {{\mathrm{diam}}}Q\le 2AC_0\delta ^k. \end{aligned}$$

### Theorem 4.7

(Whitney decomposition theorem for dyadic cubes) Let $\Omega \subsetneq X$ be open. Then there exists a pairwise disjoint collection of cubes $\mathscr {P}\subseteq \mathscr {D}^*$ such that

$$\begin{aligned} \Omega =\bigcup _{P\in \mathscr {P}}P \end{aligned}$$

and for each $P\in \mathscr {P}$,

$$\begin{aligned} {{\mathrm{diam}}}P\le d(P,\Omega ^c)\le \frac{4A^2C_0}{\gamma c_0\delta }{{\mathrm{diam}}}P. \end{aligned}$$

### Proof

We define

$$\begin{aligned} \mathscr {E}:=\left\{ Q\in \mathscr {D}^*\mid Q\subseteq \Omega ,\,{{\mathrm{diam}}}Q\le d(Q,\Omega ^c)\right\} . \end{aligned}$$

Moreover we set

$$\begin{aligned} \mathscr {P}:=\{Q\in \mathscr {E}\mid \text {there is a } k\in \mathbf {Z}\text {so that }Q\in \mathscr {D}_k,\,p(Q)\notin \mathscr {E}\}, \end{aligned}$$

where $p(Q)\in \mathscr {D}_{k-1}$ denotes the parent of $Q\in \mathscr {D}_k$. We will show that

$$\begin{aligned} \bigcup _{P\in \mathscr {P}}P=\Omega . \end{aligned}$$

Indeed, any $P\in \mathscr {P}$ is contained in $\Omega$. Conversely, if $x\in \Omega$, let $(Q^k_x)_{k\in \mathbf {Z}}$ be the sequence of cubes in $\mathscr {D}^*$ with $x\in Q^k_x$ and $Q^k_x\in \mathscr {D}_k$ for all $k\in \mathbf {Z}$. Since $\Omega$ is open, there is a ball $B=B(x;r)$ contained in $\Omega$. Picking $k_0$ large enough so that $2AC_0\delta ^{k_0}<r$, we find that

$$\begin{aligned} Q^k_x\subseteq B(x;r)\subseteq \Omega \end{aligned}$$

for all $k\ge k_0$ by (4.4). Moreover, since $d(Q^k_x,\Omega ^c)\ge A^{-1}(d(x,\Omega ^c)-2A^2C_0\delta ^k)\uparrow A^{-1}d(x,\Omega ^c)$ as $k\rightarrow \infty$, while ${{\mathrm{diam}}}(Q^k_x)\le 2AC_0\delta ^k\downarrow 0$ as $k\rightarrow \infty$, we can find a $k_1\in \mathbf {Z}$ so that ${{\mathrm{diam}}}(Q^k_x)\le d(Q^k_x,\Omega ^c)$ whenever $k\ge k_1$. Hence, for all $k\ge \max (k_0,k_1)$ we have $Q^k_x\in \mathscr {E}$. Thus, the set

$$\begin{aligned} K_x:=\left\{ k\in \mathbf {Z}\mid Q^k_x\in \mathscr {E}\right\} \end{aligned}$$

is non-empty. We also claim that $K_x$ is bounded from below. Indeed, if we choose $k_2\in \mathbf {Z}$ small enough so that $\gamma c_0\delta ^{k_2}>d(x,\Omega ^c)$, then

$$\begin{aligned} d(Q^k_x,\Omega ^c)\le d(x,\Omega ^c)<{{\mathrm{diam}}}(Q^k_x) \end{aligned}$$

for all $k\le k_2$ by (4.4), and hence $Q^k_x\notin \mathscr {E}$ for $k\le k_2$, proving the claim.

We set $k_x:=\min K_x\in \mathbf {Z}$. Then $Q^{k_x}_x\in \mathscr {E}$ while $p(Q^{k_x}_x)=Q^{k_x-1}_x\notin \mathscr {E}$. Hence, $Q^{k_x}_x\in \mathscr {P}$, proving that $x\in \cup _{P\in \mathscr {P}}P$, as desired.

Next we will show that $\mathscr {P}$ is pairwise disjoint. Suppose for a contradiction that we have $P_1,P_2\in \mathscr {P}$ so that $P_1\cap P_2\ne \emptyset$ and $P_1\ne P_2$. Let $l_1,l_2\in \mathbf {Z}$ so that $P_1\in \mathscr {D}_{l_1}$, $P_2\in \mathscr {D}_{l_2}$ and $p(P_1),p(P_2)\notin \mathscr {E}$. Without loss of generality we assume that $l_1>l_2$ and thus $P_1\subseteq P_2$ by property (ii) of the dyadic systems. Then also $p(P_1)\subseteq P_2$. Since $p(P_1)\notin \mathscr {E}$, we must have that either $p(P_1)\nsubseteq \Omega$ or $d(p(P_1),\Omega ^c)<d(p(P_1))$. The first case implies that $P_2\nsubseteq \Omega$, contradicting the fact that $P_2\in \mathscr {E}$. The second case implies that

$$\begin{aligned} {{\mathrm{diam}}}(P_2)\ge {{\mathrm{diam}}}(p(P_1))> d(p(P_1),\Omega ^c)\ge d(P_2,\Omega ^c), \end{aligned}$$

again contradicting $P_2\in \mathscr {E}$. We conclude that $\mathscr {P}$ is pairwise disjoint, as desired.

It remains to show that $d(P,\Omega ^c)<4A^2C_0/(\gamma c_0\delta ){{\mathrm{diam}}}P$ for all $P\in \mathscr {P}$. Let $P\in \mathscr {P}$, $P\in \mathscr {D}_k$ so that $p(P)\notin \mathscr {E}$. Then either $p(P)\nsubseteq \Omega$ or $d(p(P),\Omega ^c)<{{\mathrm{diam}}}(p(P))$. In the first case we have $d(p(Q),\Omega ^c)=0$, so in both cases we have

$$\begin{aligned} d(p(P),\Omega ^c)<{{\mathrm{diam}}}(p(P))\le 2AC_0\delta ^{k-1}=\frac{2AC_0}{\gamma c_0\delta }\gamma c_0\delta ^k\le \frac{2AC_0}{\gamma c_0\delta }{{\mathrm{diam}}}P. \end{aligned}$$

by (4.4). Hence,

$$\begin{aligned} d(P,\Omega ^c)\le A(d(p(P),\Omega ^c)+{{\mathrm{diam}}}(p(P)))<\frac{4A^2C_0}{\gamma c_0\delta }{{\mathrm{diam}}}P, \end{aligned}$$

as desired. $$\square $$

### Proof of Lemma 4.6

We apply the Whitney Decomposition Theorem to write $\Omega =\cup _{P\in \mathscr {P}}P$.

If $P\in \mathscr {P}$, $P\in \mathscr {D}_k$ with centre $z_P$, we have

$$\begin{aligned} 2d(z_P,\Omega ^c)&\le 2A{{\mathrm{diam}}}P+2Ad(P,\Omega ^c)\le \left( 2A+\frac{8A^3C_0}{\gamma c_0\delta }\right) {{\mathrm{diam}}}P\\&\le \left( 4A+\frac{16A^3C_0}{\gamma c_0\delta }\right) C_0\delta ^k=:\tau C_0\delta ^k \end{aligned}$$

so that

$$\begin{aligned} \emptyset \ne B(z_P;2d(z_p,\Omega ^c))\cap \Omega ^c\subseteq B(z_P;\tau C_0\delta ^k)\cap \Omega ^c. \end{aligned}$$

Since

$$\begin{aligned} \left| B(z_P;\tau C_0\delta ^k)\right| \le C\left( \frac{\tau C_0}{c_0}\right) ^\nu |B(z_P;c_0\delta ^k)|\lesssim |P| \end{aligned}$$

by (4.1), we may pick a point $x\in B(z_p;\tau C_0\delta ^k)\cap \Omega ^c$ to conclude that

$$\begin{aligned} \langle f\rangle _{1,P}\lesssim \langle f\rangle _{1,B(z_p;\tau C_0\delta ^k)}\le \mathscr {M}^{\mathscr {B}}f(x)\le \lambda . \end{aligned}$$

The assertion follows. $\square$

### Proof of Theorem 4.4

In both cases (I) and (II), the proof of Theorem 1.3 holds mutatis mutandis. Moreover, in the case (I), the same is true for Theorem 1.4, where one uses Lemma 4.6, and for Theorem 1.5, where one uses Theorem 4.7.

For Theorem 1.4 in the case (II), one replaces the set $\Omega$ in the proof by the set $\Omega =\{\mathscr {M}(|f|^{p_0})> 2[w]_{A_1} w(E)^{-1}\}$. We claim that $\Omega \ne X$. Indeed, since *X* is bounded, we have $w(X)<\infty$. Thus, by (3.1), we have

$$\begin{aligned} w(\Omega )\le \frac{w(E)}{2[w]_{A_1}}\int \!|f|^{p_0}\mathscr {M}w\,\mathrm {d}\mu \le \frac{w(E)}{2}\le \frac{w(X)}{2}<w(X), \end{aligned}$$

proving the claim. Thus we may apply Lemma 4.5 to decompose $\Omega$, and the remainder of the proof runs analogously. $\square$

## Optimality of Weighted Strong Type Estimates

In this section we are going to show that the weighted strong type estimates in (1.3) and (1.8) are optimal, given a certain asymptotic behaviour of the unweighted $L^p$ operator norm of *T*. Such asymptotic behaviour is directly linked to lower bounds on the (generalized) kernel of the operator, see Example 5.5. We improve upon the result in [6], where it was shown that the estimate (1.3) is optimal for sparse forms. Indeed, here we are directly using properties of the operator *T* itself rather than only its sparse bounds.

Our method is an adaptation of the results of Fefferman and Pipher [16] and Luque et al. [35]. We deduce sharpness of weighted bounds from the asymptotic behaviour of the unweighted $L^p$ norm of *T* as *p* tends to $p_0$ and $q_0$, respectively. The proof exploits the known sharp behaviour of the Hardy–Littlewood maximal function via the iteration algorithm of Rubio de Francia.

We will work in a doubling metric measure space $(X,d,\mu )$ satisfying the assumptions from the Sect. 4. As a matter of fact, the only property we need is a precise control of the $L^p$ norm of the maximal operator. More precisely, we let $\mathscr {D}:=\cup _{\alpha =1}^K\mathscr {D}^\alpha$ be the union of the dyadic grids in *X* obtained from Theorem 4.3. Then we define

$$\begin{aligned} \mathscr {M}_qf:=\max _{1\le \alpha \le K}\sup _{Q\in \mathscr {D}^\alpha }\langle f\rangle _{q,Q}\chi _Q=\sup _{Q\in \mathscr {D}}\langle f\rangle _{q,Q}\chi _Q \end{aligned}$$

for $1\le q<\infty$, where we set $\mathscr {M}:=\mathscr {M}_1$. Using the shorthand notation $\Vert \mathscr {M}_q\Vert _p=\Vert \mathscr {M}_q\Vert _{L^p\rightarrow L^p}$ for $p>q$, we will use

5.1 $$\begin{aligned} \Vert \mathscr {M}\Vert _p\le Kp', \end{aligned}$$

which follows as in (3.2) with $w=1$.

Let us first define the critical exponents that determine the asymptotic behaviour of the unweighted $L^p$ operator norm of *T*.

### Definition 5.1

Let $1 \le p_0 < q_0 \le \infty$. Let *T* be a bounded operator on $L^p$ for all $p_0<p<q_0$. We define

$$\begin{aligned} \alpha _T(p_0) := \sup \left\{ \alpha \ge 0 \,\mid \, \forall \varepsilon > 0, \, \limsup _{p \rightarrow p_0} \, (p-p_0)^{\alpha -\epsilon } \Vert T\Vert _{L^p\rightarrow L^p} = \infty \right\} . \end{aligned}$$

For $q_0<\infty$ we define

$$\begin{aligned} \gamma _T(q_0) := \sup \left\{ \gamma \ge 0 \,\mid \, \forall \varepsilon >0, \, \limsup _{p \rightarrow q_0} \,(q_0-p)^{\gamma -\varepsilon }\Vert T\Vert _{L^p\rightarrow L^p} = \infty \right\} , \end{aligned}$$

and for $$q_0=\infty $$

$$\begin{aligned} \gamma _T(\infty ) := \sup \left\{ \gamma \ge 0 \,\mid \, \forall \varepsilon >0, \, \limsup _{p \rightarrow \infty } \, \frac{\Vert T\Vert _{L^p\rightarrow L^p} }{ p^{\gamma -\varepsilon }} = \infty \right\} . \end{aligned}$$

For $p_0<s<q_0$ we define

$$\begin{aligned} \phi (s):=\left( \frac{q_0}{s}\right) '\left( \frac{s}{p_0}-1\right) +1. \end{aligned}$$

Then it follows from Proposition 2.1(ii) that for a weight *w* we have $w\in A_{s/p_0}\cap {{\mathrm{RH}}}_{(q_0/s)'}$ if and only if $w^{(q_0/s)'}\in A_{\phi (s)}$.

We establish the following connection between the weighted strong type estimates for *T* and the asymptotic behaviour of the unweighted $L^p$ operator norm at the endpoints $p=p_0$ and $p=q_0$.

### Theorem 5.2

Let *T* be a bounded operator on $L^p$ for all $p_0<p<q_0$. Suppose that for some $p_0<s<q_0$ and for all $w\in A_{s/p_0}\cap {{\mathrm{RH}}}_{(q_0/s)'}$,

5.2 $$\begin{aligned} \Vert T\Vert _{L^s(w) \rightarrow L^s(w)} \le c \left[ w^{(q_0/s)'}\right] _{A_{\phi (s)}}^{\beta /(q_0/s)'}. \end{aligned}$$

Then

$$\begin{aligned} \beta \ge \max \left( \frac{p_0}{s-p_0}\alpha _T(p_0),\left( \frac{q_0}{s}\right) '\gamma _T(q_0) \right) . \end{aligned}$$

We also establish a version involving the $A_1$ characteristics. Its proof follows the same lines as the one for Theorem 5.2 and will therefore be omitted.

### Theorem 5.3

Let *T* be a bounded operator on $L^p$ for all $p_0<p<q_0$. Suppose that for some $p_0<s<q_0$ and for all $w\in A_1\cap {{\mathrm{RH}}}_{(q_0/s)'}$,

5.3 $$\begin{aligned} \Vert T\Vert _{L^s(w) \rightarrow L^s(w)} \le c \left[ w^{(q_0/s)'}\right] _{A_1}^{\beta /(q_0/s)'}. \end{aligned}$$

Then

$$\begin{aligned} \beta \ge \left( \frac{q_0}{s}\right) '\gamma _T(q_0). \end{aligned}$$

### Proof of Theorem 5.2

We adapt the proof of [35], which is based on the iteration algorithm of Rubio de Francia. Let $p_0<p<s$, and define the operator $\mathcal {R}$ by

$$\begin{aligned} \mathcal {R}h = \sum _{k=0}^\infty \frac{1}{2^k} \frac{\mathscr {M}_{p_0}^k h}{\Vert \mathscr {M}_{p_0}\Vert _p^k} \end{aligned}$$

for $h \in L^p$ with $h\ge 0$. We claim that then

(A) $$h\le \mathcal {R}h$$,
(B) $$\Vert \mathcal {R}h\Vert _p \le 2 \Vert h\Vert _p$$,
(C) $$[(\mathcal {R}h)^{p_0}]_{A_1} \le 2^{p_0} \Vert \mathscr {M}\Vert _{p/p_0}$$.

Properties (A) and (B) are immediate. Note that (B) uses the assumption $p_0<p$. Property (C) can be seen as follows: By definition of $\mathcal {R}$, we have

$$\begin{aligned} \mathscr {M}_{p_0}(\mathcal {R}h) \le 2 \Vert \mathscr {M}_{p_0}\Vert _{p} \mathcal {R}h. \end{aligned}$$

Thus, using that $\Vert \mathscr {M}_{p_0}\Vert _{p} = \Vert \mathscr {M}\Vert _{p/p_0}^{1/p_0}$, we obtain for $v=\mathcal {R}h$

$$\begin{aligned} \mathscr {M}(v^{p_0}) = (\mathscr {M}_{p_0}v)^{p_0} \le (2\Vert \mathscr {M}_{p_0}\Vert _{p} v)^{p_0} =2^{p_0} \Vert \mathscr {M}\Vert _{p/p_0} v^{p_0}, \end{aligned}$$

which yields (C).

Let us now estimate $\Vert T\Vert _{L^p\rightarrow L^p}$, given (5.2). Let $f \in L^p$. Then by Hölder’s inequality,

$$\begin{aligned} \Vert Tf\Vert _p&= \left( \int |Tf|^p (\mathcal {R}|f|)^{-(s-p)\frac{p}{s}} (\mathcal {R}|f|)^{(s-p)\frac{p}{s}} \,\mathrm {d}\mu \right) ^{1/p} \\&\le \left( \int |Tf|^s (\mathcal {R}|f|)^{-(s-p)} \,\mathrm {d}\mu \right) ^{1/s} \left( \int (\mathcal {R}|f|)^p \,\mathrm {d}\mu \right) ^{\frac{s-p}{ps}}. \end{aligned}$$

We abbreviate $w:=(\mathcal {R}|f|)^{-(s-p)}$. Applying assumption (5.2) and (B) in the first step and (A) in the second step yields

$$\begin{aligned} \Vert Tf\Vert _p&\le c \left[ w^{(q_0/s)'}\right] _{A_{\phi (s)}}^{\beta /(q_0/s)'} \left( \int |f|^s w \,\mathrm {d}\mu \right) ^{1/s} \Vert f\Vert _p^{\frac{s-p}{s}} \\&\lesssim \left[ w^{(q_0/s)'}\right] _{A_{\phi (s)}}^{\beta /(q_0/s)'} \left( \int |f|^p \,\mathrm {d}\mu \right) ^{1/s} \Vert f\Vert _p^{1-\frac{p}{s}} \\&= c\left[ w^{(q_0/s)'}\right] _{A_{\phi (s)}}^{\beta /(q_0/s)'}\Vert f\Vert _p.\\&= c\left[ w^{(q_0/s)'(1-{\phi (s)}')}\right] _{A_{{\phi (s)}'}}^{({\phi (s)}-1)\beta /(q_0/s)'} \Vert f\Vert _p, \end{aligned}$$

since $[w]_{A_q} = [w^{1-q'}]_{A_{q'}}^{q-1}$. Using the equality

$$\begin{aligned} \left( \frac{q_0}{s}\right) ' \frac{s-p}{{\phi (s)} -1 } = p_0 \frac{s-p}{s-p_0} \end{aligned}$$

and Jensen’s inequality with exponent $\frac{s-p}{s-p_0} <1$, we can write

$$\begin{aligned} \left[ w^{(q_0/s)'(1-{\phi (s)}')}\right] _{A_{{\phi (s)}'}} = \left[ (\mathcal {R}|f|)^{(q_0/s)'\frac{s-p}{{\phi (s)}-1}}\right] _{A_{{\phi (s)}'}} \le \left[ (\mathcal {R}|f|)^{p_0}\right] _{A_{{\phi (s)}'}}^{\frac{s-p}{s-p_0}}. \end{aligned}$$

We thus obtain

$$\begin{aligned} \Vert Tf\Vert _p&\lesssim \left[ (\mathcal {R}|f|)^{p_0}\right] _{A_{{\phi (s)}'}}^{\beta \frac{s-p}{p_0} } \Vert f\Vert _p \le \left[ (\mathcal {R}|f|)^{p_0}\right] _{A_1}^{\beta \frac{s-p}{p_0} }\Vert f\Vert _p. \end{aligned}$$

From property (C) and (5.1) we can then deduce

$$\begin{aligned} \Vert T\Vert _{L^p\rightarrow L^p} \lesssim \Vert \mathscr {M}\Vert _{p/p_0}^{\beta \frac{s-p}{p_0}} \lesssim \left( \frac{p}{p-p_0}\right) ^{\beta \frac{s-p}{p_0}}. \end{aligned}$$

This shows

$$\begin{aligned} \limsup _{p\rightarrow p_0}(p-p_0)^{\beta \frac{s-p}{p_0}}\Vert T\Vert _{L^p\rightarrow L^p}\lesssim \limsup _{p\rightarrow p_0}p^{\beta \frac{s-p}{p_0}}<\infty \end{aligned}$$

which by definition of $\alpha _T(p_0)$ implies that $\beta \ge \frac{p_0}{s-p_0} \alpha _T(p_0)$.

Now for the behaviour for $p \rightarrow q_0$ we follow the argument of [16]. We assume $q_0<\infty$. The case $q_0=\infty$ has been treated in [35] already. We abbreviate $q:=(q_0/s)'$. Let $p_0<s<p<q_0$. We again use the iteration algorithm of Rubio de Francia, but slightly change the definition of the operator $\mathcal {R}$. This time, we define $\mathcal {R}$ by

$$\begin{aligned} \mathcal {R}h = \sum _{k=0}^\infty \frac{1}{2^k} \frac{\mathscr {M}_{q} ^k h}{\Vert \mathscr {M}_{q}\Vert _{(p/s)'}^k} \end{aligned}$$

for $h \in L^{(p/s)'}$ with $h\ge 0$. Then, just as before, we have

(A) $$ h \le \mathcal {R}h$$,
(B) $$\Vert \mathcal {R}h\Vert _{(p/s)'} \le 2 \Vert h\Vert _{(p/s)'}$$,
(C) $$[(\mathcal {R}h)^q]_{A_1} \le 2^q \Vert M\Vert _{(p/s)'/q}$$.

Let $f \in L^p$. There exists $h \in L^{(p/s)'}$ with $\Vert h\Vert _{(p/s)'} =1$ and $h \ge 0$ so that by (A)

5.4 $$\begin{aligned} \Vert Tf\Vert _p^s=\Vert |Tf|^s\Vert _{p/s}\le \int |Tf|^s h\,\mathrm {d}\mu \le \int |Tf|^s \mathcal {R}h \,\mathrm {d}\mu . \end{aligned}$$

It follows from the assumption (5.2) and (B) that

$$\begin{aligned} \int |Tf|^s \mathcal {R}h \,\mathrm {d}\mu&\le c \left[ (\mathcal {R}h)^{(q_0/s)'}\right] _{A_{\phi (s)}}^{s\beta /(q_0/s)'} \int |f|^s \mathcal {R}h \,\mathrm {d}\mu \\&\lesssim \left[ (\mathcal {R}h)^{(q_0/s)'}\right] _{A_1}^{s\beta /(q_0/s)'} \Vert f\Vert _p^s. \end{aligned}$$

Hence, by (5.4) and (C), we have

$$\begin{aligned} \Vert T\Vert _{L^p \rightarrow L^p} \lesssim \left[ (\mathcal {R}h)^{(q_0/s)'}\right] _{A_1}^{\beta /(q_0/s)'} \lesssim \Vert \mathscr {M}\Vert _{(p/s)'/(q_0/s)'}^{\beta /(q_0/s)'}. \end{aligned}$$

Using (5.1), we find

$$\begin{aligned} \Vert T\Vert _{L^p\rightarrow L^p} \lesssim \left( \frac{p(q_0-s)}{s(q_0-p)}\right) ^{\beta /(q_0/s)'} \end{aligned}$$

so that

$$\begin{aligned} \limsup _{p\rightarrow q_0}(q_0-p)^{\beta /(q_0/s)'}\Vert T\Vert _{L^p\rightarrow L^p}\lesssim \limsup _{p\rightarrow q_0}\left( \frac{p(q_0-s)}{s}\right) ^{\beta /(q_0/s)'}<\infty . \end{aligned}$$

By definition of $\gamma _T(q_0)$, this yields $\beta \ge (q_0/s)' \gamma _T(q_0)$, proving the assertion. $\square$

For the application of these results to sparsely dominated operators, we make the following observation.

### Proposition 5.4

Let $1\le p_0<q_0\le \infty$ and let $T\in S(p_0,q_0)$. Then

$$\begin{aligned} \alpha _T(p_0)\le \frac{1}{p_0},\quad \gamma _T(q_0)\le \frac{1}{q_0'}. \end{aligned}$$

### Proof

This is an immediate consequence of the fact that

$$\begin{aligned} \Vert T\Vert _{L^p\rightarrow L^p}\lesssim \left[ \left( \frac{p'}{q_0'}\right) '\right] ^{\frac{1}{q_0'}}\left[ \left( \frac{p}{p_0}\right) '\right] ^{\frac{1}{p_0}}, \end{aligned}$$

which follows from Remark 3.2. $\square$

From the above, we can deduce optimality of the weighted estimates as stated in Theorem 1.6.

### Proof of Theorem 1.6

Let $\beta$ denote the best constant in the estimate

$$\begin{aligned} \Vert T\Vert _{L^p(w) \rightarrow L^p(w)}\lesssim \left[ w^{(q_0/p)'}\right] _{A_{\phi (p)}}^{\beta /(q_0/p)'}. \end{aligned}$$

Then it follows from the result (1.3) from [6] that

$$\begin{aligned} \beta \le \max \left( \frac{1}{p-p_0},\frac{q_0-1}{q_0-p}\right) . \end{aligned}$$

Conversely, it follows from Theorem 5.2 that

$$\begin{aligned} \beta \ge \max \left( \frac{p_0}{p-p_0}\alpha _T(p_0),\left( \frac{q_0}{p}\right) '\gamma _T(q_0) \right) =\max \left( \frac{1}{p-p_0},\frac{q_0-1}{q_0-p}\right) \end{aligned}$$

proving the first result. Using Theorem 5.3, the second result follows analogously. $\square$

Let us give an example of an operator *T* for which the exponent $\gamma _T(q_0)$ is known.

### Example 5.5

Let *M* be a complete $C^\infty$ Riemannian manifold *M* of dimension $n \ge 3$. Assume that *M* is the union of a compact part and a finite number of Euclidean ends, e.g. two copies of $\mathbf {R}^n$ glued smoothly along their unit circles. Then it was shown in [9] that in the case that the number of ends is at least two, the corresponding Riesz transform *T* is bounded from $L^p(M)$ to $L^p(M;T^*M)$ if and only if $1<p<n$. More precisely, it was shown in [9, Lemma 5.1] that the kernel of *T* decays only to order $n-1$. A straightforward calculation, analogous to the classical results (see e.g. [39, p.42]), shows that this implies $\gamma _T(q_0)=\gamma _T(n)=\frac{n-1}{n}$.

## References

[1] Auscher P (2007). On necessary and sufficient conditions for
$$L^p$$-estimates of Riesz transforms associated to elliptic operators
on $$\mathbb{R}^n$$ and related estimates. Mem. Am. Math. Soc..

[2] Auscher P, Martell JM (2007). Weighted norm inequalities, off-diagonal estimates and
elliptic operators. I. General operator theory and weights. Adv. Math..

[3] Auscher P, Martell JM (2008). Weighted norm inequalities, off-diagonal estimates and
elliptic operators. IV. Riesz transforms on manifolds and weights. Math. Z..

[4] Benea, C., Bernicot, F.: Conservation de certaines propriétés à travers un contrôle épars d’un opérateur et applications au projecteur de Leray-Hopf (2017). ArXiv:1703.00228

[5] Benea C, Bernicot F, Luque T (2017). Sparse bilinear forms for Bochner Riesz multipliers
and applications. Trans. Lond. Math. Soc..

[6] Bernicot F, Frey D, Petermichl S (2016). Sharp weighted norm estimates beyond Calderón–Zygmund
theory. Anal. PDE.

[7] Björn A, Björn J (2011). Nonlinear Potential Theory on Metric Spaces, Volume 17 of EMS Tracts in
Mathematics.

[8] Blunck S, Kunstmann PC (2003). Calderón–Zygmund theory for non-integral operators and
the $$H^\infty $$ functional calculus. Rev. Mat. Iberoam..

[9] Carron G, Coulhon T, Hassell A (2006). Riesz transform and $$L^p$$-cohomology for manifolds with Euclidean ends. Duke Math. J..

[10] Coifman RR, Rochberg R (1980). Another characterization of BMO. Proc. Am. Math. Soc..

[11] Coifman, R.R., Weiss, G.: Analyse harmonique non-commutative sur certains espaces homogènes. In: Lecture Notes in Mathematics, vol. 242. Springer, Berlin/New York. Étude de certaines intégrales singulières (1971)

[12] Conde-Alonso, J.M., Culiuc, A., Di Plinio, F., Ou, Y.: A sparse domination principle for rough singular integrals (2016). ArXiv:1612.09201

[13] Cruz-Uribe D, Martell JM, Pérez C (2012). Sharp weighted estimates for classical
operators. Adv. Math..

[14] Culiuc, A., Di Plinio, F., Ou, Y.: Domination of multilinear singular integrals by positive sparse forms (2016). ArXiv:1603.05317

[15] Di Plinio, F., Hytönen, T.P., Li, K.: Sparse bounds for maximal rough singular integrals via the Fourier transform (2017). ArXiv:1706.09064

[16] Fefferman R, Pipher J (1997). Multiparameter operators and sharp weighted
inequalities. Am. J. Math..

[17] Fefferman C, Stein EM (1971). Some maximal inequalities. Am. J. Math..

[18] García-Cuerva J, Rubio de Francia JL (1985). Weighted Norm Inequalities and Related Topics, Volume 116 of
North-Holland Mathematics Studies.

[19] Grafakos L (2008). Classical Fourier analysis, Volume 249 of Graduate Texts in
Mathematics.

[20] Grafakos L (2009). Modern Fourier Analysis, Volume 250 of Graduate Texts in
Mathematics.

[21] Grigor’yan AA (1991). The heat equation on noncompact Riemannian
manifolds. Mat. Sb..

[22] Hajłasz P, Koskela P (2000). Sobolev met Poincaré. Mem. Am. Math. Soc.

[23] Hofmann S, Martell JM (2003). $$L^p$$ bounds for Riesz transforms and square roots associated to
second order elliptic operators. Publ. Mat..

[24] Hytönen TP (2012). The sharp weighted bound for general Calderón–Zygmund
operators. Ann. Math..

[25] Hytönen TP, Kairema A (2012). Systems of dyadic cubes in a doubling metric
space. Colloq. Math..

[26] Hytönen TP, Pérez C (2013). Sharp weighted bounds involving $$A_\infty $$. Anal. PDE.

[27] Hytönen TP, Pérez C, Rela E (2012). Sharp reverse Hölder property for $$A_\infty $$ weights on spaces of homogeneous type. J. Funct. Anal..

[28] Johnson R, Neugebauer CJ (1991). Change of variable results for $$A_p$$- and reverse Hölder $${\rm RH}_r$$-classes. Trans. Am. Math. Soc..

[29] Lacey MT (2017). An elementary proof of the $$A_2$$ bound. Isr. J. Math..

[30] Lerner AK (2010). A pointwise estimate for the local sharp maximal
function with applications to singular integrals. Bull. Lond. Math. Soc..

[31] Lerner, A.K., Nazarov, F.: Intuitive dyadic calculus: the basics (2015). ArXiv:1508.05639

[32] Lerner AK, Ombrosi S, Pérez C (2008). Sharp $$A_1$$ bounds for Calderón–Zygmund operators and the relationship
with a problem of Muckenhoupt and Wheeden. Int. Math. Res. Not. IMRN.

[33] Lerner AK, Ombrosi S, Pérez C (2009). Weak type estimates for singular integrals related to
a dual problem of Muckenhoupt–Wheeden. J. Fourier Anal. Appl..

[34] Li K (2017). Two weight inequalities for bilinear
forms. Collect. Math..

[35] Luque T, Pérez C, Rela E (2015). Optimal exponents in weighted estimates without
examples. Math. Res. Lett..

[36] Nazarov, F.L., Reznikov, A., Vasyunin, V., Volberg, A.: Weak norm estimates of weighted singular operators and bellman functions (2010). https://sashavolberg.files.wordpress.com/2010/11/a11_7loghilb11_21_2010.pdf

[37] Pérez C (1994). Weighted norm inequalities for singular integral
operators. J. Lond. Math. Soc..

[38] Petermichl S, Volberg A (2002). Heating of the Ahlfors–Beurling operator: weakly
quasiregular maps on the plane are quasiregular. Duke Math. J..

[39] Stein EM (1993). Harmonic Analysis: Real-Variable Methods, Orthogonality, and Integrals,
Volume 43 of Princeton Mathematical Series.

[40] Wang F-Y, Yan L (2013). Gradient estimate on convex domains and
applications. Proc. Amer. Math. Soc..

[41] Wilson JM (1987). Weighted inequalities for the dyadic square function
without dyadic $$A_\infty $$. Duke Math. J..

[42] Wilson JM (1989). Weighted norm inequalities for the continuous square
function. Trans. Am. Math. Soc..

[43] Wilson, J.M.: Weighted Littlewood–Paley theory and exponential-square integrability. In: Volume 1924 of Lecture Notes in Mathematics, Springer, Berlin (2008)

